# Chromophore Protonation State Controls Photoswitching of the Fluoroprotein asFP595

**DOI:** 10.1371/journal.pcbi.1000034

**Published:** 2008-03-21

**Authors:** Lars V. Schäfer, Gerrit Groenhof, Martial Boggio-Pasqua, Michael A. Robb, Helmut Grubmüller

**Affiliations:** 1 Department of Theoretical and Computational Biophysics, Max-Planck-Institute for Biophysical Chemistry, Göttingen, Germany; 2 Laboratoire de Chimie et Physique Quantiques, IRSAMC, Université Paul Sabatier, Toulouse, France; 3 Department of Chemistry, Imperial College London, London, United Kingdom; University of Southern California, United States of America

## Abstract

Fluorescent proteins have been widely used as genetically encodable fusion tags for biological imaging. Recently, a new class of fluorescent proteins was discovered that can be reversibly light-switched between a fluorescent and a non-fluorescent state. Such proteins can not only provide nanoscale resolution in far-field fluorescence optical microscopy much below the diffraction limit, but also hold promise for other nanotechnological applications, such as optical data storage. To systematically exploit the potential of such photoswitchable proteins and to enable rational improvements to their properties requires a detailed understanding of the molecular switching mechanism, which is currently unknown. Here, we have studied the photoswitching mechanism of the reversibly switchable fluoroprotein asFP595 at the atomic level by multiconfigurational ab initio (CASSCF) calculations and QM/MM excited state molecular dynamics simulations with explicit surface hopping. Our simulations explain measured quantum yields and excited state lifetimes, and also predict the structures of the hitherto unknown intermediates and of the irreversibly fluorescent state. Further, we find that the proton distribution in the active site of the asFP595 controls the photochemical conversion pathways of the chromophore in the protein matrix. Accordingly, changes in the protonation state of the chromophore and some proximal amino acids lead to different photochemical states, which all turn out to be essential for the photoswitching mechanism. These photochemical states are (i) a neutral chromophore, which can *trans-cis* photoisomerize, (ii) an anionic chromophore, which rapidly undergoes radiationless decay after excitation, and (iii) a putative fluorescent zwitterionic chromophore. The overall stability of the different protonation states is controlled by the isomeric state of the chromophore. We finally propose that radiation-induced decarboxylation of the glutamic acid Glu215 blocks the proton transfer pathways that enable the deactivation of the zwitterionic chromophore and thus leads to irreversible fluorescence. We have identified the tight coupling of *trans-cis* isomerization and proton transfers in photoswitchable proteins to be essential for their function and propose a detailed underlying mechanism, which provides a comprehensive picture that explains the available experimental data. The structural similarity between asFP595 and other fluoroproteins of interest for imaging suggests that this coupling is a quite general mechanism for photoswitchable proteins. These insights can guide the rational design and optimization of photoswitchable proteins.

## Author Summary

Proteins whose fluorescence can be reversibly switched on and off hold great promise for applications in high-resolution optical microscopy and nanotechnology. To systematically exploit the potential of such photoswitchable proteins and to enable rational improvements of their properties requires a detailed understanding of the molecular switching mechanism. Here, we have studied the photoswitching mechanism of the reversibly switchable fluoroprotein asFP595 by atomistic molecular dynamics simulations. Our simulations explain measured quantum yields and excited state lifetimes, and also predict the structures of the hitherto unknown intermediates and of the irreversibly fluorescent state. Further, we find that the proton distribution in the active site of the asFP595 controls the photochemical conversion pathways of the chromophore in the protein matrix. Our results show that a tight coupling between *trans-cis* isomerization of the chromophore and proton transfer is essential for the function of asFP595. The structural similarity between asFP595 and other fluoroproteins suggests that this coupling is a quite general mechanism for photoswitchable proteins. These insights can guide the rational design and optimization of photoswitchable proteins.

## Introduction

Fluorescent proteins have been widely used as genetically encodable fusion tags to monitor protein localizations and dynamics in live cells [1]–[3]. Recently, a new class of green fluorescent protein (GFP)-like proteins has been discovered, which can be reversibly photoswitched between a fluorescent *(on)* and a non-fluorescent *(off)* state [4]–[10]. As the reversible photoswitching of photochromic organic molecules such as fulgides or diarylethenes is usually not accompanied by fluorescence [11], this switching reversibility is a very remarkable and unique feature that may allow fundamentally new applications. For example, the reversible photoswitching, also known as kindling, may provide nanoscale resolution in far field fluorescence optical microscopy much below the diffraction limit [12]–[15]. Likewise, reversibly switchable fluorescent proteins will enable the repeated tracking of protein location and movement in single cells [16]. Since fluorescence can be sensitively read out from a bulky crystal, the prospect of erasable three-dimensional data storage is equally intriguing [17].

The GFP-like protein asFP595, isolated from the sea anemone *Anemonia sulcata*, is a prototype for a reversibly switchable fluorescent protein. The protein can be switched from its non-fluorescent *off* state to the fluorescent *on* state by green light of 568 nm wavelength [5],[6],[18],[19]. From this so-called kindled *on* state, the same green light elicits a red fluorescence emission at 595 nm. Upon kindling, the intensity of the absorption maximum at 568 nm diminishes, and an absorption peak at 445 nm appears. The kindled *on* state can be promptly switched back to the initial *off* state by this blue light of 445 nm. Alternatively, the *off* state is repopulated through thermal relaxation within seconds. In addition, if irradiated with intense green light over a long period of time, asFP595 can also be irreversibly converted into a fluorescent state that cannot be quenched by light any more [5]. The nature of this state is hitherto unknown.

The switching cycle of asFP595 is reversible and can be repeated many times without significant photobleaching. These properties render asFP595 a promising fluorescence marker for high-resolution optical far-field microscopy, as recently demonstrated by Hofmann and coworkers [20]. Currently, however, with its low fluorescence quantum yield (<0.1% and 7% before and after activation, respectively [6],[16]) and rather slow switching kinetics, the photochromic properties of asFP595 need to be improved. To systematically exploit the potential of such switchable proteins and to enable rational improvements to the properties of asFP595, a detailed molecular understanding of the photoswitching mechanism is mandatory.

The aim of this study is to obtain a detailed mechanistic picture of the photoswitching mechanism of asFP595 at the atomic level, i.e., to understand the dynamics of both the activation process (*off*-to-*on* switching) and the de-activation process (*on*-to-*off* switching).

**Figure 1:**
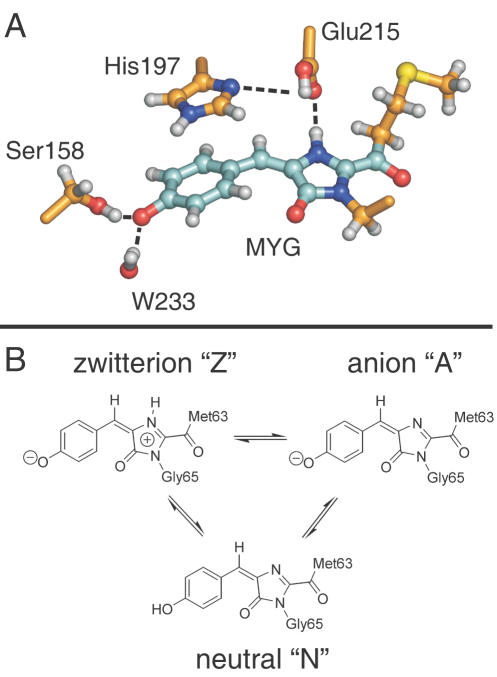
(A) Chromophore (MYG) in the *trans* conformation and adjacent amino acid side chains. Ser158, Glu215, and the crystallographic water molecule W233 are hydrogen-bonded to MYG (dashed lines). His197 is π-stacked to the MYG phenoxy-moiety and forms a hydrogen bond to Glu215 (dashed line). The carbon skeleton of the quantum mechanical (QM) subsystem is shown in cyan, and the carbon atoms modelled by molecular mechanics (MM) are shown in orange. (B) Schematic drawings of the different MYG protonation states considered in this work.

High-resolution crystal structures of the wild-type (wt) asFP595 in its *off* state [19],[21],[22], of the Ser158Val mutant in its *on* state [19], and of the Ala143Ser mutant in its *on* and *off* states [19] were recently determined. Similar to GFP, asFP595 adopts a β-barrel fold enclosing the chromophore, a 2-acetyl-5-(*p*-hydroxybenzylidene)imidazolinone (Figure 1). The chromophore is post-translationally formed in an autocatalytic cyclization-oxidation reaction of the Met63-Tyr64-Gly65 (MYG) triad. As compared to the GFP chromophore, the π-system of MYG is elongated by an additional carbonyl group [23].

Reversible photoswitching of asFP595 was possible even within protein crystals, and x-ray analysis showed that the *off-on* switching of the fluorescence is accompanied by a conformational *trans-cis* isomerization of the chromophore [19]. In a recent study [24], we have shown that the isomerization induces changes of the protonation pattern of the chromophore and some of the surrounding amino acids, and that these changes account for the observed shifts in the absorption spectrum upon kindling. Based on the comparison between measured and calculated absorption spectra, the major protonation states in the ground state have been assigned to the zwitterion (Z) and the anion (A) for the *trans* conformer, whereas the neutral (N) chromophore is dominant for the *cis* conformation (Figure 1B).

Here, we study the photochemical behavior of each of the previously identified protonation states. We have addressed the following questions: How does light absorption induce the isomerization of the chromophore within the protein matrix, and how do the different protonation states affect the internal conversion mechanism? Which is the fluorescent species, and how can the fluorescence quantum yield be increased? To address these questions, we have carried out nonadiabatic molecular dynamics (MD) simulations using a hybrid quantum-classical QM/MM approach. This approach includes diabatic surface hopping between the excited state and the ground state. The forces acting on the chromophore were calculated using the CASSCF [25],[26] multi-reference method, which, although not always yielding highly accurate excitation and fluorescence energies, has shown to be a reliable method for mechanistic studies of photochemical reactions involving conical intersections [27].

A number of approaches for modeling nonadiabatic dynamics have been described in the literature, such as Tully's fewest switches surface hopping [28], and multiple spawning [29]. For recent reviews, see [30],[31]. In the context of QM/MM simulations, the surface hopping approach to photobiological problems has been pioneered by Warshel and coworkers [32],[33]. The diabatic surface hopping approach used in this work differs from the other approaches in two main respects. First, in our approach a binary decision (hop or no hop) is made at each integration time step of the trajectory, based only on the current wavefunctions of the ground and excited states. Second, hopping is only allowed at the conical intersection (CI) seam, where hopping probability approaches unity. This could in principle underestimate the crossing probabilty, because we do not allow for transitions in regions of strong coupling but no real crossing. However, for ultra-fast photochemical reactions in large polyatomic systems, decay predominantly takes place at the CI seam, as also shown by others [31]. Thus, most surface hops are essentially diabatic, justifying our approach. In addition, both energy and momentum are conserved upon a transition, as the trajectory never leaves the diabatic energy surface. The theoretical background and algorithmic implementation of the diabatic surface hopping are detailed in the Supporting Information (Text S4).

Several theoretical studies on the photochemistry of the GFP chromophore have been conducted, applying both static ab initio [34]–[36] and DFT calculations [37], and dynamics simulations based on a semi-empirical Hamiltonian [38]. In addition, vertical excitation energies of asFP595 model chromophores in the gas phase and in a continuum dielectric were calculated by DFT and ab initio methods [39],[40], as well as in a minimal protein environment by means of DFT and CASSCF calculations within a QM/MM approach [41].

By identifying key residues in the cavity of the asFP595 chromophore, our nonadiabatic QM/MM molecular dynamics simulations elucidate how the protein surrounding governs the photoreactivity of this photoswitchable protein. Based on the simulations, we provide a new mechanism that qualitatively explains measured decay times and quantum yields, and that predicts the structures and protonation states of the photochemical intermediates and of the irreversibly fluorescent state. We also suggest excited state proton transfer (ESPT) to play an important mechanistic role. However, the detailed study of such ESPT processes is beyond the scope of this paper. Our predictions can be probed by, e.g., time-resolved Fourier transform infrared (FTIR) spectroscopy and x-ray crystallography.

## Results/Discussion

Our results reveal that the excited state behavior of asFP595 is determined by the protonation pattern of the chromophore and some amino acids in the surrounding protein matrix rather than by the chromophore conformation (*trans* or *cis*). The latter, however, modulates the excited state properties by changing the hydrogen-bonded network in the chromophore cavity.

For both conformers, we identified three possible species that differ in protonation state, explaining the complex photochemical behavior of asFP595. First, the neutral chromophores N_trans_ and N_cis_ undergo reversible *trans-cis* photoisomerization and thus account for the photoswitching between the dark *off* and fluorescent *on* states. Second, anionic chromophores A_trans_ and A_cis_ lead to the observed ultra-fast radiationless deactivation. Third, fluorescence emission can in principle originate from both zwitterions Z_trans_ and Z_cis_. The protonation states are interchangeable *via* proton transfers. In the following we will describe the excited state behavior of all three protonation states.

### *trans-cis* Isomerization of the Neutral Chromophore

**Figure 2:**
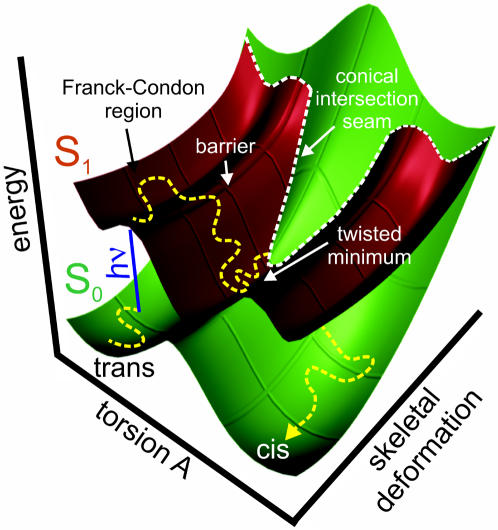
Schematic representation of potential energy surfaces. The excited (S_1_, red) and ground (S_0_, green) states of the neutral chromophore are shown along the *trans-cis* isomerization coordinate (torsion A) and a skeletal deformation coordinate of the chromophore. Radiationless decay occurs at the S_1_/S_0_ conical intersection (CI, dashed white line). In this representation, the CI occurs as an extended seam, because the torsion coordinate is from the (N-2)-dimensional intersection space, and the skeletal deformation coordinate is from the 2-dimensional branching pace. The dashed yellow line represents the path sampled in a QM/MM photoisomerization trajectory.

The five excited state simulations that were initiated from the ground state trajectory of the *trans* neutral chromophore N_trans_ are listed in Table 1. *Trans*-to-*cis* photoisomerization of the chromophore was observed in one of these simulations (run b, Table 1; Video S2 in Supporting Information). Figure 2 shows a schematic representation of the S_0_ (green) and S_1_ (red) potential energy surfaces of the neutral chromophore, along with a photoisomerization MD trajectory (yellow dashed line). Two coordinates are shown, the isomerization coordinate and a skeletal deformation coordinate of the chromophore (see below). The dynamics can be separated into three distinct phases: (i) evolution on the electronic ground state S_0_, (ii) excitation and evolution on the excited state S_1_, and (iii) decay back to S_0_ at the surface crossing seam followed by subsequent relaxation on the ground state surface. The position of the surface crossing seam controls the passage of the trajectory from S_1_ to S_0_. The seam is accessed from a global twisted minimum on S_1_, which is separated by a small S_1_ barrier from a local planar minimum near the Franck-Condon (FC) region.

**Table 1:** Excited state lifetimes and final conformations from the MD simulations initiated in the neutral *trans* chromophore conformation.

Starting in N_trans_		
Run	S_1_ lifetime (ps)	Final conformation
a	0.516	*trans*
b	0.475	*cis*
c	0.309	*trans*
d	0.224	*trans*
e	0.718	*trans*

In our simulations, individual excited state (S_1_) lifetimes between 0.224 ps and 0.718 ps were observed (Table 1). A simple exponential fit to the observed lifetimes yielded a decay time of τ = 0.34 ps (σ_+_ = 0.21 ps, σ_−_ = 0.13 ps, with σ being the statistical error). Given the low number of trajectories, the statistical error of our estimated lifetime may seem unexpectedly low, but results from a rigorous analysis assuming an underlying single exponential decay [42]. Recent femtosecond time-resolved pump/probe experiments by Schüttrigkeit and coworkers have yielded excited state decay time constants of 0.32 ps (78%), 2.6 ps (19%), and 12.1 ps (3%) as well as a fluorescence lifetime of 2.2 ns for asFP595 [43]. However, although the simulated decay times seem to be in good agreement to the experimental results, we believe that the results should not be directly compared. In previous work [24], we demonstrated that the N_trans_ protonation state is hardly populated in asFP595 and therefore is unlikely to contribute to the observed excited state decay. Instead, the species that is predominantly responsible for the ultra-fast radiationless decay observed in the experiments is the anionic *trans* chromophore A_trans_, as shown in detail below.

**Figure 3:**
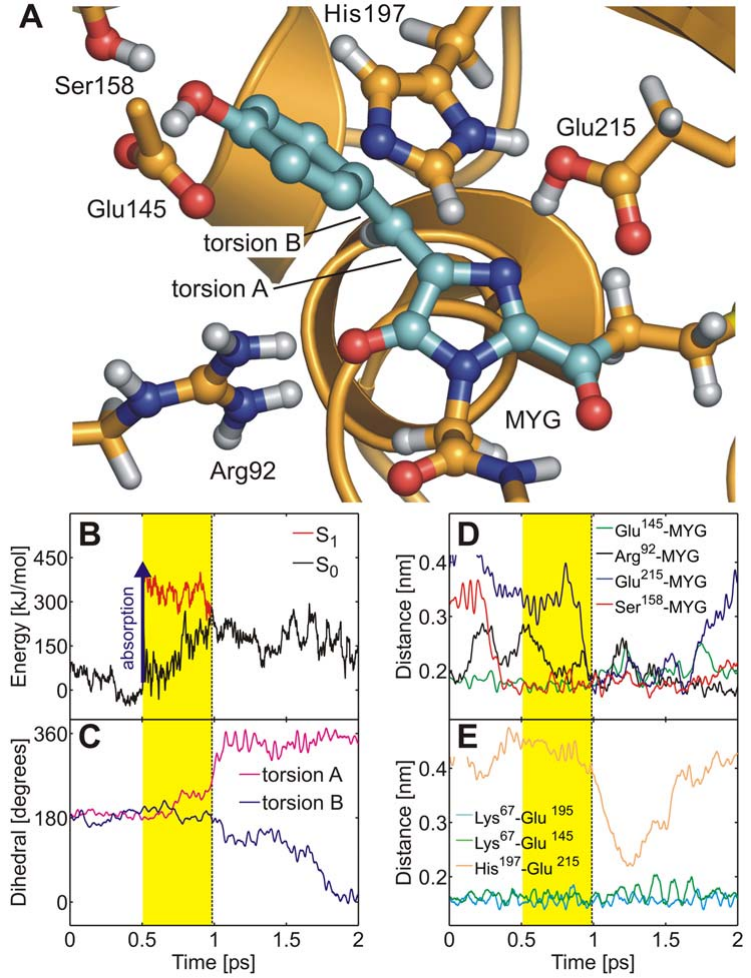
*Trans*-*cis* isomerization of the neutral chromophore. (A) Chromophore (MYG) conical intersection geometry adopted during the MD simulation. MYG forms hydrogen bonds to Arg92, Glu145, Ser158, and Glu215. Color code as in Figure 1. (B) Ground (S_0_, black) and excited (S_1_, red) potential energy traces along the QM/MM molecular dynamics trajectory. Photon absorption (blue arrow) excites the chromophore into S_1_ (yellow area) until it decays back to S_0_ at the conical intersection seam (dashed line). (C) Time-evolution of the ring-bridging torsion angles A (magenta) and B (blue). (D, E) Change of the hydrogen bonding network in the chromophore cavity during *trans*-*cis* isomerization. The MYG-Arg92 (black), MYG-Glu145 (green), MYG-Ser158 (red), Lys67-Glu195 (cyan, residues not shown in (A)), and Lys67-Glu145 (green) hydrogen bonds were stable during isomerization. Additional hydrogen bonds between MYG and Glu215 (blue) as well as between His197 and Glu215 (orange) were transiently formed.

Figure 3A shows the snapshot from the isomerization trajectory (run b, Table 1) shortly before the surface crossing seam was encountered.

After photon absorption (blue arrow in Figures 2 and 3B), the chromophore spontaneously rotated around torsion A (imidazolinone-twist), and the ring-bridging CH group pointed downwards (away from His197) by almost 90°. The time-evolution of the S_0_ and S_1_ potential energies and of the two ring-bridging torsion angles during *trans*-*cis* photoisomerization are shown in Figures 3B and 3C. After excitation to S_1_, the chromophore rapidly relaxed from the FC region into a nearby planar S_1_ minimum, as is evident from the decreasing S_1_ energy in panel b (red curve). The system stayed in this planar minimum for about 0.2 ps; subsequently the global twisted S_1_ minimum was reached through rotation around torsion A (Figures 2, 3B, and 3C). The system then oscillated around this minimum until the conical intersection seam was encountered. After the surface hop to S_0_, torsion B (hydroxyphenyl-twist) rotated after a short delay of about 0.5 ps. Previously, an ideal “hula-twist” isomerization mechanism of the zwitterionic chromophore was proposed based on a force field model [19]. This hula-twist involves a *simultaneous* rotation around *both* torsion angles A and B. In the QM/MM simulations of the neutral chromophore presented here, rotation around both torsions was also observed. However, twisting around torsion B was slower than around torsion A (Figure 3C), and the isomerization hence proceeded *via* the twisted conformer shown in Figure 3A, with perpendicular imidazolinone and hydroxyphenyl moieties. The hula-twist isomerization mechanism of the zwitterion at the QM/MM level will be discussed below.

During the initial equilibration of N_trans_, the hydrogen bonding network of the x-ray crystal structures of the anionic and zwitterionic chromophores (see above) changed to accommodate the non-native neutral chromophore. First, a stable hydrogen bond formed between the hydroxyphenyl OH group of MYG and Glu145 (Figure 3A). Second, the hydrogen bonds between the imidazolinone nitrogen and Glu215 as well as between His197 and Glu215 ruptured. Interestingly, these two hydrogen bonds were transiently re-established during the end of the second isomerization phase (blue and orange curves in Figures 3D and 3E, respectively) in which torsion B followed torsion A. In the twisted intermediate structure, the imidazolinone nitrogen atom was sterically more exposed as compared to the planar conformation, which facilitated the formation of the hydrogen bonds. During an extended 10 ns force field simulation, the His197-Glu215 and MYG-Glu215 hydrogen bonds repeatedly broke and re-formed at a timescale of several hundred picoseconds (data not shown), further underlining the flexibility of these two hydrogen bonds. Figures 3D and 3E furthermore show that during the isomerization, the hydrogen bonding network in the chromophore cavity was stable, as none of the hydrogen bonds that were established at the instant of photoexcitation ruptured. A similar stability was found in all simulations, irrespective of the chromophore conformation or protonation state. Thus, the hydrogen bonding network around the chromophore is flexible enough to allow for photoexcitation and even photoisomerization without being ruptured.

For the neutral *cis* chromophore N_cis_, five excited state simulations were initiated from the ground state trajectory (Table 2). Only two of these trajectories returned to the ground state within 10 ps (Table 2, runs a and b), which was the maximum affordable trajectory length in terms of computation time. In one of these two simulations, a spontaneous *cis*-to-*trans* photoisomerization was observed (run a; Video S1 in Supporting Information). As expected, the isomerization pathway was similar to the reverse *trans*-to-*cis* pathway in that the conical intersection seam was accessed *via* rotation around torsion A, followed by a slightly delayed rotation around torsion B in S_0_ (see Figure S4 in Supporting Information). However, in contrast to the activation pathway, the ring-bridging CH group rotated upwards (i.e., towards His197). Thus, despite the anisotropic protein surrounding, both rotational orientations of the chromophore CH bridge are feasible. In the second simulation, the CI seam was also encountered after rotation around torsion A, but the chromophore returned to the initial *cis* conformation.

**Table 2:** Excited state lifetimes and final conformations from the MD simulations initiated in the neutral *cis* chromophore conformation.

Starting in N_cis_		
Run	S_1_ lifetime (ps)	Final conformation
a	0.374	*trans*
b	3.561	*cis*
c*	1.573	*trans*
d*	0.867	*cis*
e*	1.206	*trans*

*: In runs c, d, and e, the escape from the S_1_ minimum was accelerated by conformational flooding.

For the other three trajectories, the chromophore remained trapped in a planar S_1_ minimum conformation near the FC region throughout the simulation (data not shown in Table 2). The starting structures of these trajectories were used for three additional simulations in which the escape from the planar S_1_ minimum was accelerated by means of conformational flooding [42],[44] (Table 2, runs c–e). In these accelerated simulations, the flooding potential successfully induced the escape from the local S_1_ minimum, and the surface crossing seam was encountered in all cases. Isomerization was observed in two of these simulations. In total, *cis*-to-*trans* photoisomerization was seen in three out of five simulations initiated in the N_cis_ state. Although the number of trajectories is small, our simulations suggest that the probability for *cis*-to-*trans* photoisomerization is larger than for the reverse *trans*-to-*cis* process discussed before.

### S_1_/S_0_ Conical Intersection Topology and S_1_ Minima

To further characterize the potential energy surfaces underlying the photochemical conversion processes of the neutral chromophore (Figure 2), we have optimized three S_1_ minima and a minimum energy S_1_/S_0_ conical intersection (MECI) in the gas phase (see Supporting Information, Figure S1, Tables S1 and S5). We found a local planar S_1_ minimum for the *trans* isomer 76 kJ/mol below the FC region. The structure corresponding to the global S_1_ minimum is twisted around torsion A by 85° and lies −131 kJ/mol relative to the FC region. The structure of the nearby MECI is twisted around torsion A by 81°. The MECI is energetically lower than the FC region by 62 kJ/mol, and the CI seam is therefore readily accessible. Twisting around torsion B instead of torsion A also leads to a local minimum on S_1_, whose energy is 28 kJ/mol below the FC energy.

The MD simulations reflect this surface topology. Immediately after excitation, the system relaxed from the FC region to the global S_1_ minimum by rotation around torsion A (Figure 3C). The system oscillated around this minimum until the conical intersection seam was encountered, with a subsequent surface hop back to S_0_. The gradients on S_0_ and S_1_ are almost parallel at the CI, which indicates that the CI is sloped. The gradient difference vector and the derivative coupling vector that span the branching space largely correspond to skeletal deformations of the imidazolinone moiety (see Supporting Information, Figure S1). Thus, as shown in Figure 2, the rotation coordinate around torsion A is parallel to the seam and does not lift the S_1_/S_0_ degeneracy. The seam is accessible anywhere along this torsional rotation coordinate, and therefore such torsional rotation is in principle not essential for the radiationless decay. The extended surface crossing seam parallel to the isomerization coordinate accounts for the low isomerization quantum yield seen in our simulations. In most of our MD simulations, the seam was encountered rather “early” along the torsional rotation coordinate (Figure 2), and the system thus returned to the ground state before overcoming the S_0_ barrier maximum. In these cases, relaxation on S_0_ after the surface hop led back to the starting conformation.

### Role of the Protein Environment

To elucidate the influence of the protein environment on the photoisomerization process of the chromophore, we have re-calculated the S_1_ and S_0_ energies along two excited state trajectories (run b, Table 1 and run a, Table 2) in the gas phase. In these simulations, the chromophore followed the same trajectory as before, but did not interact with the rest of the system (protein and solvent surrounding). We have not attempted to further characterize the electrostatic influence of the surrounding by, *e.g.*, pK_a_ calculations.

**Figure 4:**
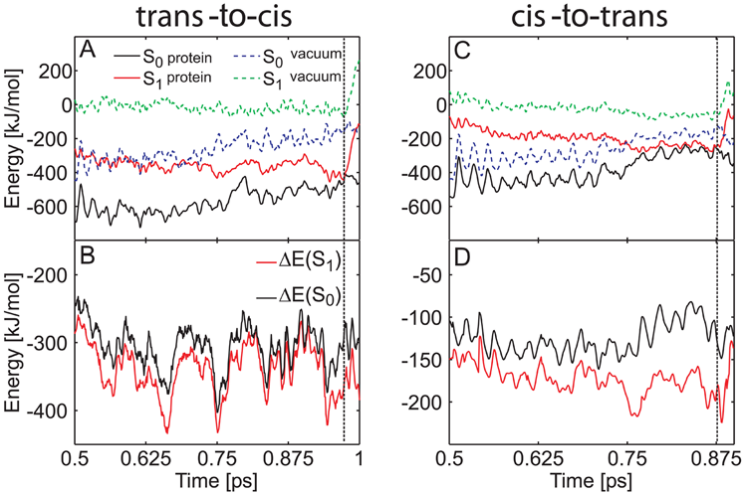
Influence of the protein environment on the photoisomerization process of the neutral asFP595 chromophore. (A, C) Ground and excited state energies along *trans*-to-*cis* (A) and *cis*-to-*trans* (C) isomerization trajectories (run b, Table 1 and run a, Table 2). The protein environment stabilizes S_0_ and S_1_ (black and red lines, respectively) relative to the gas phase (dashed blue and green lines, respectively). (B, D) Energy difference between the protein and the gas phase. ΔE(S_0_) = E(S_0_, protein)−E(S_0_, gas phase) is plotted in black, ΔE(S_1_) = E(S_1_, protein)−E(S_1_, gas phase) in red. The protein environment energetically stabilizes S_1_ more strongly than S_0_. The vertical dashed black line represents the surface crossing. The energy offset in (A) and (C) is 1.9699×10^6^ kJ/mol.

Figure 4A and 4C show the obtained energy traces. In the protein, both S_1_ and S_0_ are stabilized with respect to the gas phase. For the *trans*-to-*cis* isomerization process, the protein stabilized the energies of the S_1_ and S_0_ states on average by −339 kJ/mol and −307 kJ/mol, respectively. For the *cis*-to-*trans* process, the average stabilization energies were −173 kJ/mol and −126 kJ/mol, respectively. Thus, the protein (and solvent) environment favors S_1_ over S_0_ by about 30–50 kJ/mol. Figure 4B and 4D show the energy differences between the protein and the gas phase, ΔE = E(protein)−E(gas phase). The S_1_ stabilization was rather strong at the surface crossing seam (Figure 4). We found S_1_ to be stabilized stronger than S_0_ by 78 kJ/mol and 93 kJ/mol at the conical intersection in both MD simulations. In summary, the protein environment energetically stabilizes S_1_ more than S_0_, thereby enhancing fast radiationless decay.

### Ultra-Fast Radiationless Deactivation of the Anionic Chromophore

In total, 20 simulations of the anionic chromophore protonation state were carried out, 10 of which were initiated in the *trans* conformation and the other 10 were initiated in the *cis* conformation. Ultra-fast radiationless deactivation was observed in all 20 trajectories (Table S6 in Supporting Information). However, *trans-cis* photoisomerization never occurred. A simple exponential fit to the S_1_ lifetimes of the *trans* anion yielded a decay time of τ = 0.45 ps (σ_+_ = 0.19 ps, σ_−_ = 0.12 ps). Since A_trans_ is one of the two dominant protonation states in the *off* state besides Z_trans_ [24], we expect A_trans_ to significantly contribute to the experimentally observed decay. The measured decay time of 0.32 ps [43] agrees well with the decay time derived from the simulations. For A_cis_, an excited state decay time of τ = 1.81 ps (σ_+_ = 0.77 ps, σ_−_ = 0.48 ps) was obtained, which is about four times longer as compared to the decay time of A_trans_.

**Figure 5:**
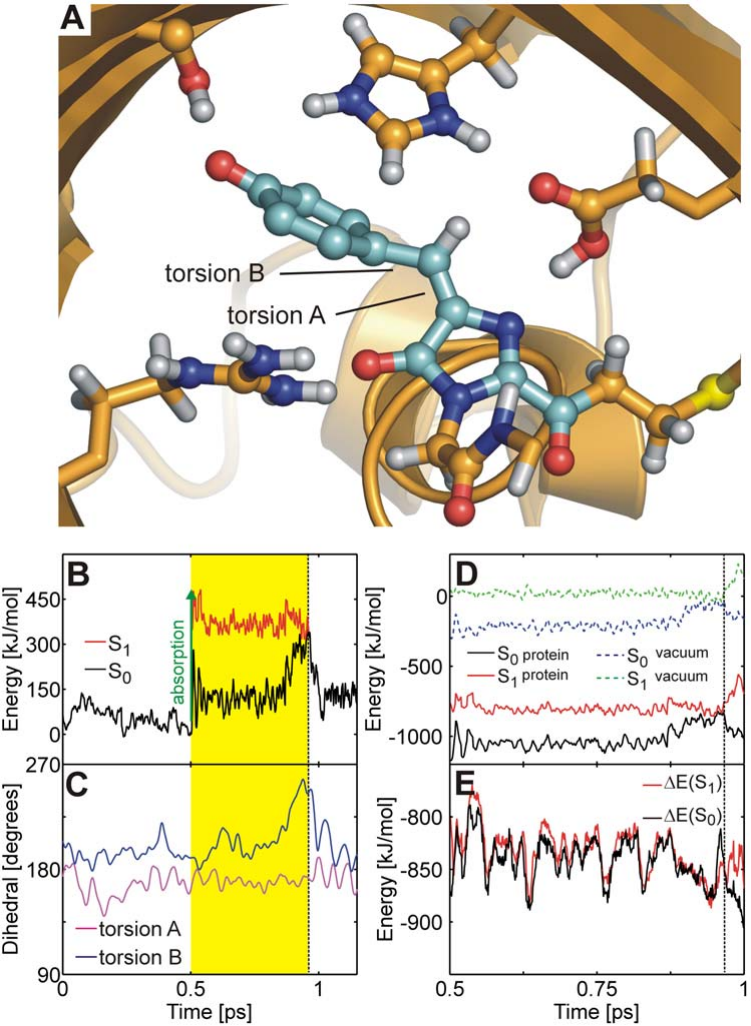
Ultra-fast internal conversion mechanism of the *trans* anion. (A) Snapshot at the conical intersection: the chromophore is twisted around torsion B, yet the hydrogen bonded network in the chromophore cavity remains intact. (B) Ground (S_0_, black) and excited (S_1_, red) potential energy traces along the QM/MM molecular dynamics trajectory. Photon absorption (green arrow) brings the chromophore into S_1_ (yellow area) until it decays back to S_0_ at the conical intersection seam (dashed line). (C) Time-evolution of the torsion angles A (magenta) and B (blue). (D) S_0_ and S_1_ energies along a representative excited state trajectory of A_trans_. The protein environment strongly stabilizes S_0_ and S_1_ (black and red lines, respectively) relative to the gas phase (dashed blue and green lines, respectively). The energy offset is 1.9686×10^6^ kJ/mol. (E) Energy difference ΔE between the protein and the gas phase for S_0_ (black) and S_1_ (red).

Figure 5A shows the conical intersection geometry adopted during a typical trajectory. In contrast to the neutral chromophore, the CI seam was accessed through a phenoxy-twist (rotation around torsion B, see Figure 5C), and the CH bridge remained in the imidazolinone plane. Shortly after excitation, rotation around torsion B drove the system towards the surface crossing seam (Figure 5B and 5C). Back on S_0_, the system returned to the initial configuration. The hydrogen bonding network in the chromophore cavity was very similar to the network observed in the x-ray crystal structures and remained stable during the excited state MD simulations.

Since rotation around torsion B does not lead to *trans-cis* isomerization and rotation around torsion A did not occur, the quantum yield for the isomerization of the anion was zero in our simulations. However, due to the limited number of trajectories (20), we cannot rule out the *trans-cis* photoisomerization of the anion. Our results agree with recent MRPT2 computations of Olsen and coworkers on an anionic DsRed-like model chromophore in the gas phase [45], who have shown that the imidazolinone-twisted S_1_/S_0_ CI (i.e., twisted around torsion A), which leads to *cis-trans* isomerization, is disfavored by more than 150 kJ/mol as compared to the phenoxy-twisted CI. The latter CI is lower in energy than the planar S_1_ minimum by 9.2 and 48.1 kJ/mol at the MRPT2 and CASSCF levels, respectively [45]. In our calculations, this energy difference is about 30 kJ/mol (see Supporting Information, Table S2), in qualitative agreement with Olsen and coworkers. Due to the slightly different chromophores in DsRed and asFP595, a quantitative agreement cannot be expected.

The deviation from planarity of the *trans* chromophore observed in the crystal structures was speculated to enhance ultra-fast deactivation [24],[43]. Indeed, the difference between the S_1_ lifetimes of the *cis* and *trans* conformers seen in our simulations can be attributed to steric constraints imposed by the protein matrix. In the *trans* conformation the phenoxy-ring deviates from planarity by about 20°, whereas the *cis* chromophore is essentially planar [19],[24]. We observed in our simulations that only a slight additional twisting was required for the *trans* conformer to reach the surface crossing seam. Thus, the pre-twisting of the phenoxy-moiety due to the protein matrix facilitated fast internal conversion of the *trans* conformer.

### S_1_/S_0_ Conical Intersection Topology and S_1_ Minima

As shown in Supporting Information (Figure S2, Table S2), we have optimized the S_1_/S_0_ MECI, a planar and two twisted S_1_ minima (imidazolinone-twist and phenoxy-twist) for an isolated anionic chromophore. The planar minimum lies 31 kJ/mol below the FC point. The structure of the global S_1_ minimum is twisted around torsion B by 269° and its energy lies −82 kJ/mol relative to the FC energy. The MECI structure is also twisted about torsion angle B by 269° and is energetically lower than the FC point by 61 kJ/mol, thus explaining the ultra-fast decay seen in our MD simulations. Twisting around torsion A leads to a local S_1_ minimum that is 46 kJ/mol below the FC point. The CI of the anion is sloped, and the gradient difference vector corresponds to a skeletal deformation of the imidazolinone ring, analogous to the neutral chromophore (see above). In contrast to the neutral chromophore, the derivative coupling vector involves rotation around torsion B. However, the amplitude of this vector is small. Thus, the two electronic states may remain close in energy along torsion B, allowing the system to decay at various phenoxy-twist angles.

### Role of the Protein Environment

To study the influence of the protein matrix on the deactivation process of the anionic chromophore, we have re-evaluated the S_0_ and S_1_ energies along two representative excited state trajectories (*trans* and *cis*) with all interactions between the QM atoms of the chromophore and the MM surrounding switched off, as was done for the neutral chromophores (see above). As Figure 5D and 5E show, the protein (and solvent) environment stabilizes the chromophore with respect to the gas phase. The S_0_ and S_1_ states of the *trans* chromophore are strongly stabilized by −840 kJ/mol and −832 kJ/mol, respectively. The S_0_ and S_1_ states of the *cis* chromophore were stabilized relative to the gas phase by −407 kJ/mol and −433 kJ/mol, respectively, during a representative A_cis_ trajectory (Figure S5 in Supporting Information). Similar to the neutral chromophore, the protein environment favors the *trans* conformation over *cis*. Before reaching the CI seam, the S_0_ and S_1_ states of the chromophore were stabilized to the same extent. At the CI, however, the protein environment lowered the energy of the S_1_ state more strongly than the energy of the S_0_ state by 26 kJ/mol and 20 kJ/mol for A_trans_ and A_cis_, respectively. This preferential stabilization of S_1_ enhanced the ultra-fast radiationless deactivation seen in our MD simulations.

### Fluorescence Emission of the Zwitterionic Chromophores

Ten simulations were carried out for the zwitterion. Five simulations were started in the Z_trans_ conformation, and the other five simulations were initiated in the Z_cis_ conformation. No decay back to the ground state was observed within a maximum trajectory length of 10 ps, neither for Z_trans_ nor for Z_cis_. The chromophore did not escape from a planar S_1_ minimum in the vicinity of the FC region in any of the excited state simulations. This suggests that Z_trans_ and Z_cis_ could be the fluorescent species in asFP595, although the measured fluorescence lifetime of 2.2 ns [43] is still much longer than our maximal trajectory length (10 ps).

For Z_trans_ and for Z_cis_, we have carried out three additional simulations each, in which we applied the conformational flooding technique [42],[44],[46] to accelerate the escape from the S_1_ minimum in an unbiased manner (see Materials and Methods). The results of these flooding simulations are shown in the Supporting Information (Figure S6, Text S3). The flooding potential induced isomerization of the chromophore, which followed a hula-twist pathway, in agreement with our previous work [19]. From the flooding simulations, we obtained a lower bound for the excited state lifetime of the order of 1 ns. The qualitative agreement with the measured fluorescence decay time of 2.2 ns provides further support for the assignment of the zwitterion as the fluorescent species. The results thus obtained for the zwitterionic chromophore suggest that a hula-twist CI may be spontaneously accessed if the trajectories were extended to (significantly) longer times, i.e., nanoseconds. However, at the nanosecond timescale, fluorescence (and not isomerization) will be the predominant decay process. Note that in Ref. [19], the energy barrier for hula-twist isomerization of the zwitterion was underestimated, thus favoring this isomerization over fluorescence. In the next paragraph, we characterize the CI and show that the minimum energy crossing point for the zwitterionic chromophore has a high energy, thus hampering radiationless decay through hula-twist isomerization.

### S_1_/S_0_ Conical Intersection Topology and Influence of the Protein Environment

To characterize the topology of the S_1_ and S_0_ potential energy surfaces and of the conical intersection that occurs between them, multiconfigurational calculations were carried out for the isolated zwitterionic chromophore, as was also done for the other protonation states (see above). We optimized a planar S_1_ minimum and a hula-twist S_1_/S_0_ MECI (see Supporting Information, Figure S3, Tables S3 and S4). In contrast to the anion and the neutral chromophore, no twisted S_1_ minima were found. The gradient difference vector and the derivative coupling vector at the MECI do not involve torsional rotation of either torsion A or torsion B, indicating that the CI seam lies parallel to the isomerization coordinate. The MECI lies 70 kJ/mol above the planar S_1_ minimum and 23.4 kJ/mol above the FC energy. Hence, in contrast to the anion and the neutral chromophore, no low-lying CI is present for the zwitterion, demonstrating that radiationless decay in the gas phase cannot occur in an unactivated manner. For the CI seam to become accessible, a significant stabilization of S_1_ relative to S_0_ by the protein environment would be required. However, as shown in Figure S6 in Supporting Information, the protein surrounding does not reduce the S_1_/S_0_ energy gap anywhere along the isomerization coordinate.

### Deactivation of Z_trans_ through Proton Transfer

Our results suggest that the zwitterionic chromophore is potentially fluorescent, irrespective of the conformation. However, the x-ray analysis of the emitting species has shown that only the *cis* chromophore fluoresces, whereas the *trans* chromophore is dark [19]. A possible explanation for this discrepancy is the presence of an alternative deactivation channel that does not involve isomerization. This deactivation pathway would have to be more easily accessible for Z_trans_ than for Z_cis_. Only the latter would therefore be trapped in S_1_ and fluoresce.

The hydrogen bond between the NH group of the imidazolinone ring and Glu215 strongly suggests that the alternative decay involves an excited state proton transfer (ESPT). Such ESPT would quench the fluorescence, because the resulting anion rapidly deactivates, as shown above. However, by including only the chromophore into the QM subsystem, we have excluded the possibility of observing such ESPT in our QM/MM simulations.

**Figure 6:**
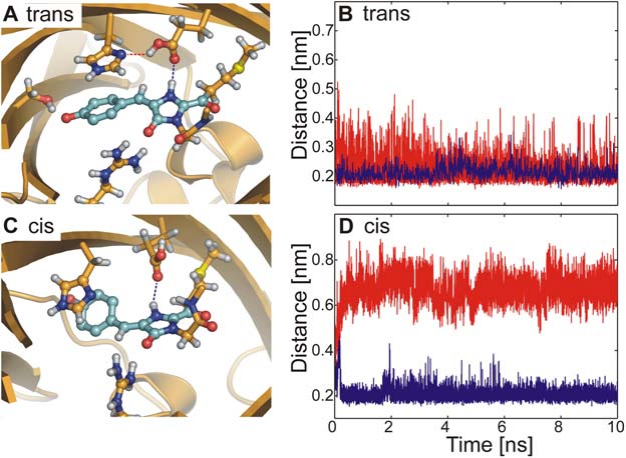
Hydrogen bonding network in the chromophore cavity during force field simulations of zwitterionic chromophores. (A, C) Snapshots from MD simulations of Z_trans_ and Z_cis_, respectively. The blue dashed lines indicates the distance between the NH proton of MYG and Glu215, and the red dashed line that between the OH-group of Glu215 and the N_δ_ atom of His197. (B, D) Time-evolution of the two hydrogen bonds shown in (A) and (C) during representative force field MD simulations.

To identify possible ESPT pathways, we have carried out extended force field MD simulations of both Z_trans_ and Z_cis_ and analyzed the relevant hydrogen bonds. Figure 6A shows that, during the simulation of Z_trans_, two stable hydrogen bonds were formed between the protonated OH group of Glu215 and His197 as well as between the NH proton of MYG and Glu215. These two hydrogen bonds allow for a proton transfer from Z_trans_ to the rapidly deactivating A_trans_. The OH proton of Glu215 could transfer to the N_δ_ atom of His197, with a simultaneous or subsequent transfer of the NH proton of the imidazolinone moiety to Glu215. In contrast, during the force field simulation of Z_cis_, the MYG-Glu215 hydrogen bond remained intact, whereas the Glu215-His197 hydrogen bond broke after about 1 ns (Figure 6B). This differential behavior of Z_trans_ and Z_cis_ was confirmed by two additional independent MD simulations (data not shown). Based on these results, we assume that only the *trans* zwitterion can be converted to the anion through a short proton wire. Therefore, an ultra-fast deactivation channel is available only for the *trans* zwitterion, and not for the fluorescent *cis* zwitterion. From the presence of the hydrogen bonding network in our force field trajectories, we do not obtain insights into the energetics of proton transfer. Studying these transfers along the identified pathways in asFP595, both in the ground and the excited state, is beyond the scope of the present work.

**Figure 7:**
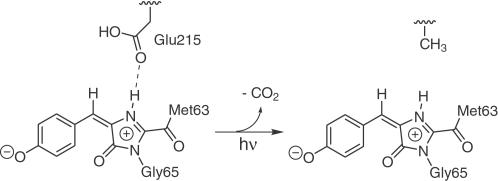
Scheme of the proposed decarboxylation of Glu215, which yields an irreversibly fluorescent zwitterion.

Having established that fluorescence can only originate from the zwitterionic chromophores, the structure of the irreversibly fluorescent state of asFP595 can now be predicted. We expect that intense irradiation over a prolonged period of time leads to a decarboxylation of the Glu215 side chain (Figure 7). Such process is also known to occur in GFP [47],[48] and DsRed [49]. A decarboxylated Glu215 can no longer take up the NH proton from the zwitterionic chromophores. The absence of an S_1_ ESPT deactivation channel leads to fluorescence. The finding that the irreversibly fluorescent state cannot be switched *off* by light (see Introduction) is corroborated by our observation that even in the flooding-induced isomerization trajectories, no radiationless decay back to S_0_ occurred (see Figure S6 in Supporting Information).

### Switching Efficiency of asFP595

**Figure 8:**
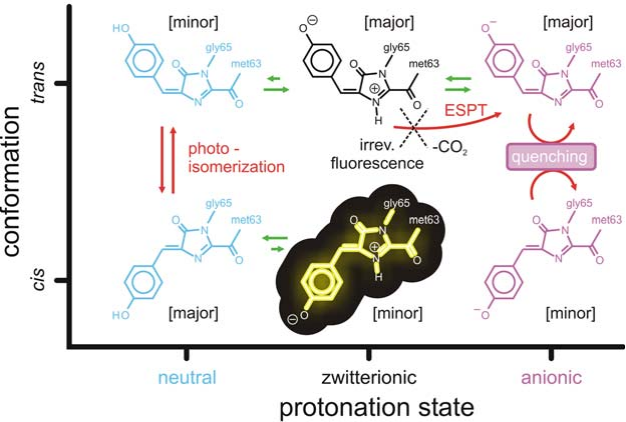
Scheme of the reversible photoswitching mechanism of asFP595 proposed in this work. The fluorescent state Z_cis_ is highlighted. The green arrows indicate ground state equilibria, whereas the red arrows indicate excited state processes. The major protonation states are the zwitterionic and the anionic chromophores in the *trans* conformation, and the neutral chromophore in the *cis* conformation, as indicated in the square brackets.

Figure 8 summarizes our proposed photoswitching mechanism. The proton distribution at the active site of asFP595 governs the photochemical conversion pathways of the chromophore in the protein matrix. Changes in the protonation state of the chromophore and several proximal amino acids lead to different photochemical states, which are all involved in the photoswitching process. These photochemical states are (i) the neutral chromophores N_trans_ and N_cis_, which can undergo *trans-cis* photoisomerization, (ii) the anionic chromophores A_trans_ and A_cis_, which rapidly undergo radiationless decay after excitation, and (iii) the potentially fluorescent zwitterions Z_trans_ and Z_cis_. The overall stability of the different protonation states is controlled by the isomeric state of the chromophore.

To switch from the non-fluorescent *off* to the fluorescent *on* state, the chromophore has to isomerize from *trans* to *cis*. As shown in Figure 8, this photoisomerization was only observed for the neutral form of the chromophore (N_trans_→ N_cis_). However, the N_trans_ state is only marginally populated [24], thus explaining the low quantum yield for switching asPF595 to the *on* state. Moreover, green light is used to switch on asFP595, whereas the absorption maximum of N_trans_ is significantly blue-shifted. The use of blue light, however, would lead to an unfavorable N_cis_→ N_trans_ back-reaction due to the absorption of the blue light by N_cis_.

The reverse *cis*-to-*trans* isomerization, i.e., *on*-to-*off* switching, requires the excitation of the neutral *cis* chromophore. Since N_cis_ is the predominant protonation state in the *cis* conformation, the efficiency for the *on*-to-*off* switching is high, as observed experimentally [19]. Fluorescence originates from Z_cis_, which is also hardly populated, like N_trans_ [24]. Taken together, the low populations of the involved states give rise to the low overall fluorescence quantum yield of asFP595.

The insight obtained from our simulations can be exploited for a targeted improvement of asFP595 for applications as a fluorescence marker in optical microscopy. In particular, to improve the signal-to-noise ratio, a higher fluorescence quantum yield is desired. Our results suggest that one way to enhance fluorescence would be to increase the stability of Z_cis_, e.g., by introducing additional hydrogen bond donors near the phenoxy-group of the chromophore. Another possibility would be to implement an internal proton relay, similar to that in GFP. In GFP, a hydroxyphenyl-bound serine residue, a water molecule, and a glutamic acid form an internal proton wire that enhances the formation of the fluorescent A_cis_ chromophore from the neutral chromophore *via* ESPT. GFP has a significantly higher fluorescence quantum yield as compared to asFP595 [50]–[54]. Although the fluorescent species in asFP595 and GFP are different, the similarity between the chromophores suggests that implementing a similar internal proton relay in asFP595 might increase its fluorescence quantum yield.

Note, however, that due to the competition between different reaction channels in asFP595, shifting the relative populations of the protonation states will also affect the photoswitchability. For example, increasing the population of the fluorescent species at the cost of the neutral species will decrease the back-isomerization efficiency. Thus, a compromise has to be found between increasing the fluorescence quantum yield on the one hand while maintaining the photoswitchability of asFP595 on the other hand.

### Conclusions

Understanding the excited state dynamics of ultra-fast photoactivated processes in biomolecular systems such as the reversible photoswitching of the fluorescent protein asFP595 represents a major challenge, but is essential to unveil the underlying molecular mechanisms. In the present work we have demonstrated that by using an *ab initio* QM/MM excited state molecular dynamics strategy together with explicit surface hopping, it is not only possible to explain at the atomic level experimentally accessible quantities of asFP595, such as quantum yields and excited state lifetimes, but also to make predictions that are rigorously testable by experiment, such as the nature of the irreversibly fluorescent state or possible improvement of the fluorescence quantum of this protein.

We have revealed that the protonation pattern of the chromophore cavity determines the photochemical behavior of asFP595, and that photon absorption can lead to *trans-cis* isomerization of the neutral chromophore. Based on our results, we suggest that a reversibly switchable protein must fulfill three criteria. First, to enable switching, *trans-cis* photoisomerization is necessary. Second, this photoisomerization has to be coupled to proton transfer events, that is, the preferred protonation state is different for the two conformers. Third, only one of the two isomers fluoresces, while the other can undergo rapid radiationless decay.

Interestingly, other fluoroproteins contain a chromophoric moiety similar to asFP595, like, e.g., Dronpa [8],[55],[56], DsRed [49],[57], Kaede or KiKG [58],[59], eqFP611 [60]–[63], Rtms5 [64], and HcRed [65]. In these structures *cis* or *trans* conformations of the chromophore have been observed. Our simulations on asFP595 suggest that chromophore photoisomerization could also be possible in these fluoroproteins. In particular, the high structural similarity between asFP595 and the reversibly photoswitchable Dronpa protein suggests that the Dronpa chromophore can undergo *trans-cis* photoisomerization as well. Indeed, the crystal structure of the isomerized state of Dronpa was solved very recently [66] and confirms our prediction. Furthermore, recent experiments on Dronpa [67],[68] also provide strong support for our proposed protonation/deprotonation mechanism. The similarity between the chromophores in a variety of fluoroproteins suggests that during molecular evolution, the (*p*-hydroxybenzylidene)imidazolinone chromophoric moiety served as a template and that the photochromic properties - and thus the function - was fine-tuned by the protein environment.

## Materials and Methods

To model the dynamics of the photoactivated asFP595 chromophore, we carried out excited state QM/MM [69] molecular dynamics (MD) simulations. Energies and forces of the excited (S_1_) and ground (S_0_) states were calculated on-the-fly at the CASSCF/3-21G level of theory with a reduced active space of 6 electrons in 6 orbitals. The studied processes start in S_1_ and end up in S_0_. The transitions (hops) between the two energy surfaces were modeled by surface selection at the conical intersection (CI) seam (see Supporting Information, Text S4 for details). This diabatic hopping procedure is known to potentially underestimate the surface hopping probability [70],[71]. However, our experience has shown that in large polyatomic systems, low-lying CI hyperlines are easily accessed due to their high dimensionality (N−2, where N is the number of internal degrees of freedom). We therefore expect that, for the case at hand, diabatic surface hopping is sufficiently accurate. For all MD simulations, Gromacs 3.3 [72] with an interface [70] to Gaussian03 [73] was used. Modifications were made to the one-electron integral routines of Gaussian03 to account for the polarization of the CASSCF wavefunctions due to the pointcharges on the MM atoms.

The reduced active space used in the MD simulations was validated using geometry optimizations of relevant excited state minima and minimum energy crossing points for the isolated chromophores. The full CASSCF active space for the π-system of the asFP595 chromophore would require 18 π electrons in 16 π orbitals, rendering geometry optimizations prohibitively expensive. To make the respective calculations feasible, we have restricted the number of excitations in the wavefunction by employing the RASSCF method (see Supporting Information, Text S1) [74]–[76]. We have characterized minima and conical intersections at the RASSCF(18,7+4+5)[2,2]/6-31G* level of theory. The final 6 electron, 6 orbital active space used in the CASSCF QM/MM MD simulations was selected from the RASSCF calculations such as to enable the simultaneous description of the electronic ground and first excited states. This approach allowed us to compute on-the-fly QM/MM trajectories using a CASSCF/3-21G wavefunction with an accuracy that is comparable to RASSCF/6-31G* at a drastically reduced computational cost.

All MD simulations were based on the crystal structure of a mutant of asFP595 [24]. The mutant has similar photochromic properties as the wild-type, but high-resolution crystal structures are available for both the *trans* and the *cis* conformations. For both *trans* and *cis* isomers, MD simulations were initiated for three different chromophore protonation states; anionic (A), neutral (N), and zwitterionic (Z). The simulations were performed in a rectangular periodic box of about 730 nm^3^. Each system contained about 21,500 TIP4P water molecules, including 340 crystallographic water molecules. After assigning the protonation pattern of the chromophore pocket, all other polar, aromatic, and aliphatic hydrogen atoms were added to the protein with the HB2MAK [77] routine of WHATIF [78]. To each of the systems, sodium and chloride ions were added at physiological concentration to compensate for the net positive charge of the protein. The actual number of ions varied with the total charge of the protein, which differed for the chosen protonation patterns of the chromophore cavity. The final systems comprised about 90,000 atoms.

Prior to the MD simulations, the systems were energy minimized (1000 steps steepest descent). Subsequently, force field based MD simulations were carried out. First, 500 ps MD simulations with harmonic position restraints on all protein heavy atoms (force constant 1000 kJ mol^−1^ nm^−2^) were carried out to equilibrate the solvent and the ions. Then, 500 ps free MD simulation were run at 300 K. All simulations were carried out using the OPLS all-atom force field [79]. Parameters for the chromophore were taken from [19].

The simulations were run at constant temperature and pressure by coupling to an external heat bath (τ_T_ = 0.1 ps, τ_p_ = 1 ps) [80]. In the force field simulations, LINCS [81] was used to constrain bond lengths, thus allowing a time step of 2 fs. SETTLE [82] was applied to constrain the internal degrees of freedom of the water molecules. A twin-range cut-off was used for the Lennard-Jones interactions. Interactions within 1.0 nm were updated at every step, whereas interactions between 1.0 nm and 1.6 nm were updated every ten steps. Coulomb interactions within 1.0 nm were computed at each step as well. Beyond this cut-off, the particle-mesh Ewald (PME) method [83] with a grid spacing of 0.12 nm was used.

The QM subsystem in the excited state QM/MM simulations comprised the chromophore; the rest of the system was described by the OPLS all-atom force field (Figure 1). The N−C_α_ bond of Gly65 and the C_α_−C_β_ bond of Met63, respectively, were replaced by a constraint, and the QM part was capped with hydrogen link atoms [84]. The forces on the link atoms were distributed over the two heavy atoms at the boundary according to the lever rule. The QM system experienced the Coulomb field of all MM atoms within a sphere of 1.6 nm, and Lennard-Jones interactions between QM and MM atoms were included. For the QM/MM simulations, a time step of 1 fs was used, and no constraints were applied in the QM subsystem. Prior to the excited state simulations, the systems were simulated in the ground state, first for 1 ps at the RHF/3-21G//OPLS level of theory and then for an additional 2.5 ps at the CASSCF(6,6)/3-21G//OPLS level. From the latter trajectory, frames at equal time intervals (Δt = 0.5 ps) were used as starting configurations for the excited state MD simulations.

To accelerate the escape from the S_1_ minima, additional excited state conformational flooding [42],[44] simulations were carried out for the N_cis_, Z_cis_, and Z_trans_ systems, respectively. A Gaussian-shaped flooding potential V_fl_ was constructed from a principal component analysis (PCA) [85]–[87] of free QM/MM S_1_ simulations. For all systems, the covariance matrix of the motion of all QM atoms, except the exocyclic carbonyl group of the chromophore and the hydroxyphenyl-OH proton (for N_cis_), was computed from two independent 10 ps S_1_ trajectories. All internal degrees of freedom of the chromophore were affected by the flooding potential to ensure that the escape from the initial minimum was accelerated in an unbiased manner. Unless stated differently, adaptive flooding [42],[46] with target destabilization free energies of 300 kJ/mol (Z_trans_) or 100 kJ/mol (N_cis_) and time constants of τ = 0.1 ps was applied. In all flooding simulations, V_fl_ was switched off as soon as the conical intersection seam was encountered to allow for an unperturbed relaxation on the ground state potential energy surface.

In all our simulations of the anionic and zwitterionic chromophores, we considered both the cationic and neutral protonation states of the imidazole side chain of His197, which lies coplanar to the chromophore (Figure 1). Recent Poisson-Boltzmann electrostatics calculations have shown that slight structural fluctuations in the local environment change the preferred protonation of the His197 imidazole ring between cationic and neutral and that both protonation states are populated at room temperature [24]. Thus, we ran the same number of trajectories with a cationic and with a neutral His197. As shown in Supporting Information (Text S2), the His197 protonation state had no major influence on the decay mechanisms and lifetimes.

## Supporting Information

Figure S1 Optimized ab initio geometries of N_trans_.
(0.53 MB MPG)

Figure S2 Optimized ab initio geometries of A_trans_.
(0.48 MB DOC)

Figure S3 Optimized ab initio geometries of Z_trans_.
(0.42 MB DOC)

Figure S4 *Cis*-to-*trans* isomerization of the neutral chromophore. (A) Chromophore (MYG) conical intersection geometry adopted during excited state QM/MM MD simulation. The CH bridge rotated upwards (i.e., towards His197). (B) Time-evolution of the ring-bridging dihedral angles A (magenta) and B (blue). (C) S_0_ (black) and S_1_ (red) potential energy traces along the QM/MM trajectory. Photon absorption brings the system into S_1_ (yellow area), until decay back to S_0_ occurs (dashed line) at the CI seam. (D,E) The hydrogen-bonding network in the chromophore cavity stayed stable during the isomerization of MYG.
(8.79 MB DOC)

Figure S5 Influence of the protein environment on the deactivation of the anionic *cis* chromophore A_cis_. (A) S_0_ and S_1_ energies along a representative trajectory (run h, Table S6). The protein environment stabilized S_0_ and S_1_ (black and red lines, respectively) relative to the gas phase (dashed blue and green lines, respectively). (B) Energy difference ΔE between the protein and the gas phase for S_0_ (black) and for S_1_ (red). The dashed line indicates the decay at the CI seam.
(1.30 MB TIF)

Figure S6 Isomerization of the *cis* zwitterion induced by conformational flooding. (A) Hula-twist structure adopted during isomerization trajectory (B) Ground (S_0_, black) and excited state (S_1_, red) potential energy traces along the trajectory. S_0_ and S_1_ come energetically close, but the surface crossing seam was not encountered. The surface hop was therefore imposed at the structure with the minimum energy gap (dashed black line). The time evolution of the flooding potential V_fl_ is shown in the inset. (C) Time evolution of the torsion angles A (magenta) and B (blue). (D) S_0_ and S_1_ energies (black and red lines, respectively) along the isomerization trajectory. The protein environment stabilizes S_0_ and S_1_ relative to the gas phase (dashed blue and green lines, respectively). The energy offset is 1.97·10^6^ kJ/mol. (E) Energy difference ΔE between the protein and the gas phase for S_0_ (black) and S_1_ (red). S_0_ is stabilized slightly stronger than S_1_ along the whole isomerization pathway.
(7.52 MB TIF)

Table S1 RASSCF(18,7+4+5)2,2/6-31G* results on N_trans_.
(0.03 MB TIF)

Table S2 RASSCF(18,7+4+5)2,2/6-31G* results on A_trans_.
(0.03 MB DOC)

Table S3 RASSCF(18,7+4+5)2,2/6-31G* results on Z_trans_.
(0.03 MB DOC)

Table S4 CASSCF(6,6)/3-21G results on Z_trans_.
(0.03 MB DOC)

Table S5 Cartesian coordinates of optimized structures at RASSCF(18,7+4+5)2,2/6-31G* level.
(0.05 MB DOC)

Table S6 Excited state lifetimes and conformations from the MD simulations of the anionic chromophores A_trans_ and A_cis_. In runs A–J, His197 was modelled as cationic, whereas in runs K-T, His197 was modelled as neutral (singly protonated at N_δ_).
(0.04 MB DOC)

Text S1 Ab initio calculations in the gas phase.
(0.03 MB DOC)

Text S2 Influence of the π-stacked Histidine 197.
(0.02 MB DOC)

Text S3 Conformational flooding simulations of the zwitterionic chromophores.
(0.02 MB DOC)

Text S4 Diabatic Surface Hopping: Theory and Implementation.
(0.06 MB DOC)

Video S1 *cis*-to-*trans* isomerization of neutral chromophore.
(24.36 MB DOC)

Video S2 *trans*-to-*cis* isomerization of neutral chromophore.
(51.31 MB MPG)
