# Supplementary material for: Chromophore Protonation State Controls Photoswitching of the Fluoroprotein asFP595
Source: PLoS Comput Biol. 2008 Mar 21;4(3):e1000034. doi: 10.1371/journal.pcbi.1000034 (PMC2274881; doi:10.1371/journal.pcbi.1000034)

**Optimized *ab initio* geometries of N*_trans_***

**S_0_ planar minimum:**


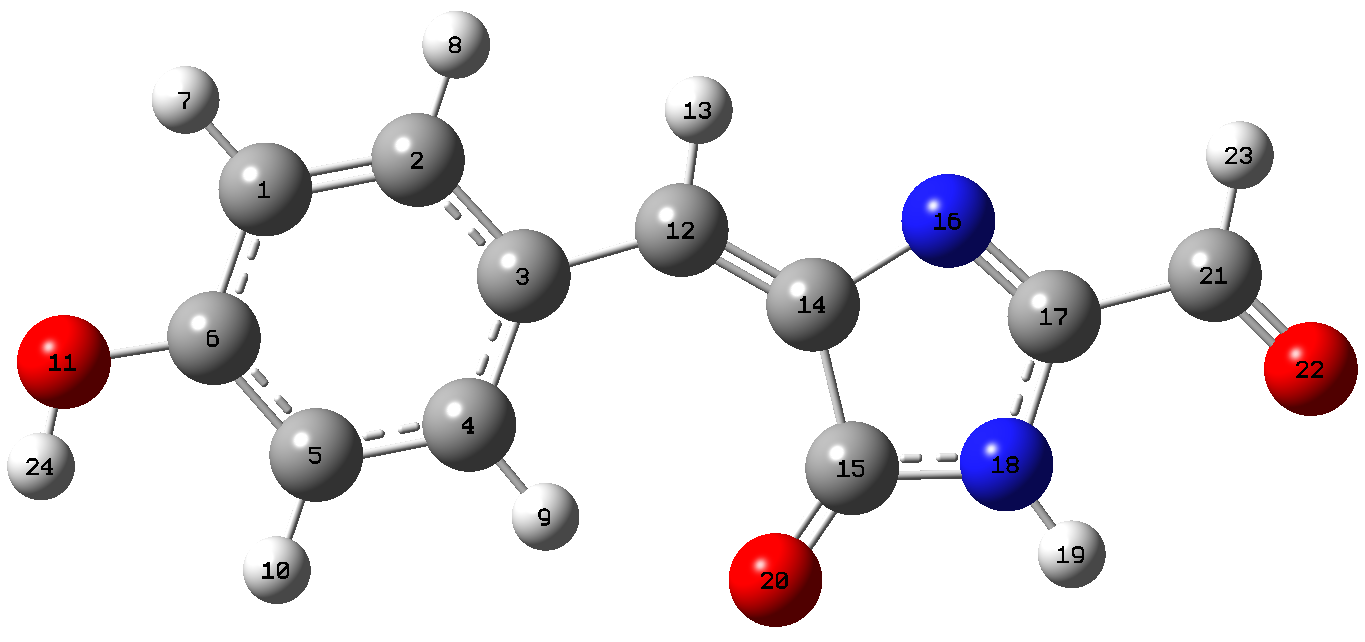


1.38

1.39

1.39

1.39

1.35

1.41

1.40

1.46

1.35

1.41

1.28

1.50

1.38

1.20

1.38

1.47

1.20

**S_1_ planar minimum:**


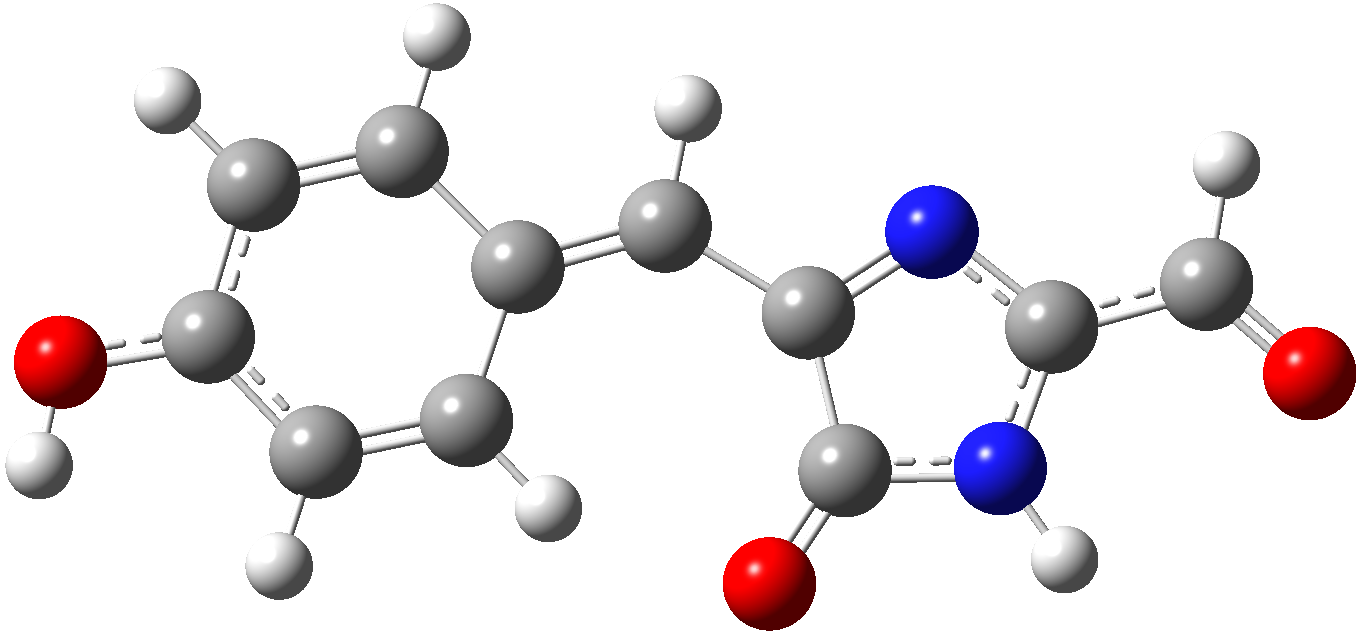


1.35

1.37

1.43

1.41

1.33

1.46

1.46

1.36

1.49

1.32

1.36

1.45

1.38

1.22

1.35

1.43

1.21

**S_1_ minimum torsion A (imidazolinone twist):**


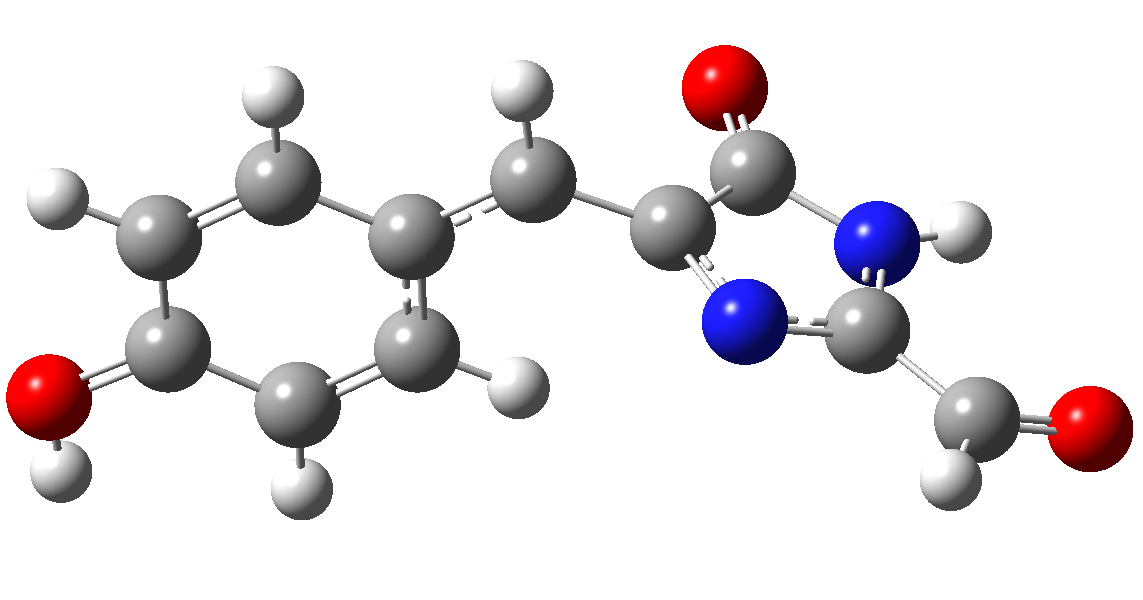


1.32

1.41

1.42

1.37

1.43

1.37

1.43

1.39

1.46

1.36

1.30

1.37

1.39

1.22

1.42

1.44

1.21

**S_1_ minimum torsion B (phenyl twist):**


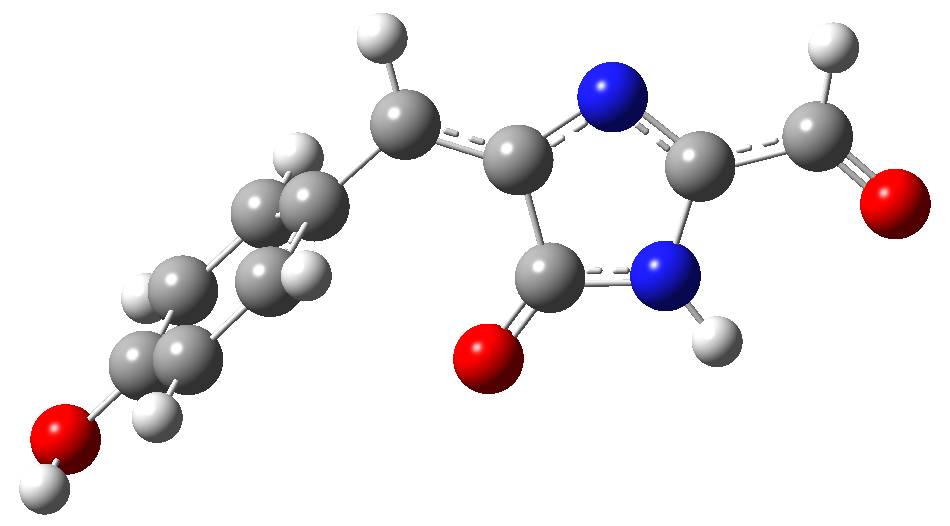


1.31

1.41

1.42

1.36

1.36

1.44

1.43

1.47

1.39

1.47

1.22

1.36

1.39

1.43

1.22

1.32

1.35

**S_1_/S_0_ one-bond flip MECI:**

**
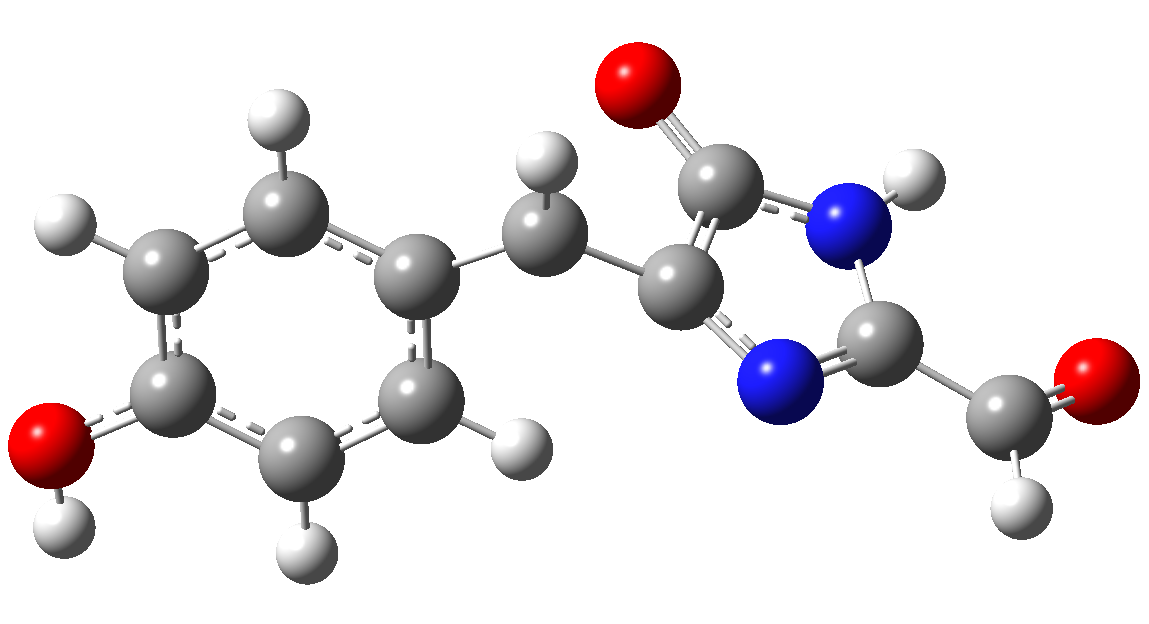
**

1.39

1.38

1.41

1.40

1.35

1.41

1.43

1.44

1.48

1.34

1.38

1.34

1.36

1.27

1.31

1.44

1.20

**Derivative coupling vector at S_1_/S_0_ MECI:**


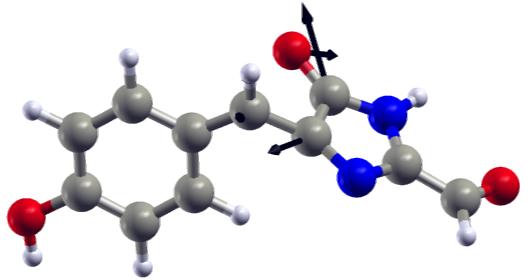


**Gradient difference vector at S_1_/S_0_ MECI:**


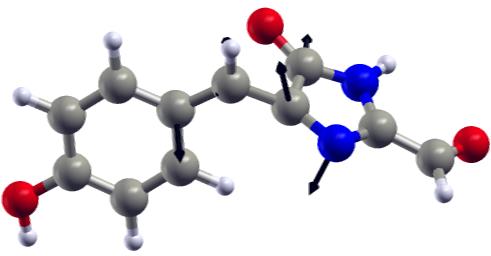


**S_0_ gradient at S_1_/S_0_ MECI:**


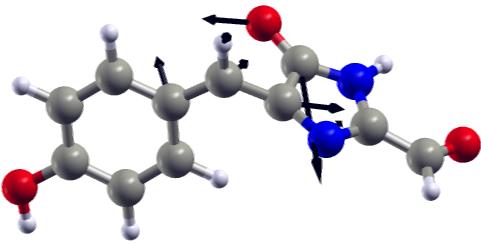


**S_1_ gradient at S_1_/S_0_ MECI:**


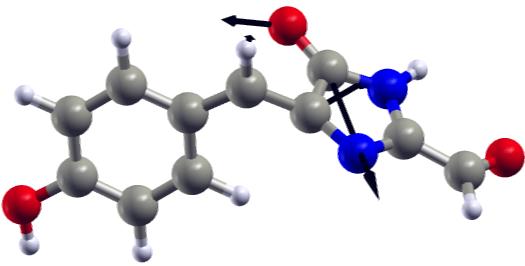

Supplement: Figure S2 — Optimized ab initio geometries of Atrans. (0.48 MB DOC) [file pcbi.1000034.s002.doc]
