# Supplementary material for: Chromophore Protonation State Controls Photoswitching of the Fluoroprotein asFP595
Source: PLoS Comput Biol. 2008 Mar 21;4(3):e1000034. doi: 10.1371/journal.pcbi.1000034 (PMC2274881; doi:10.1371/journal.pcbi.1000034)
Supplement: Figure S3 — Optimized ab initio geometries of Ztrans. (0.42 MB DOC) [file pcbi.1000034.s003.doc]

**Optimized *ab initio* geometries of A*_trans_***

**S_0_ planar minimum:**

1.22

1.46

1.35

1.45

1.38

1.41

1.38

1.46

1.35

1.45

1.45

1.22

1.38

1.38

1.30

1.21

1.44

**S_1_ planar minimum:**


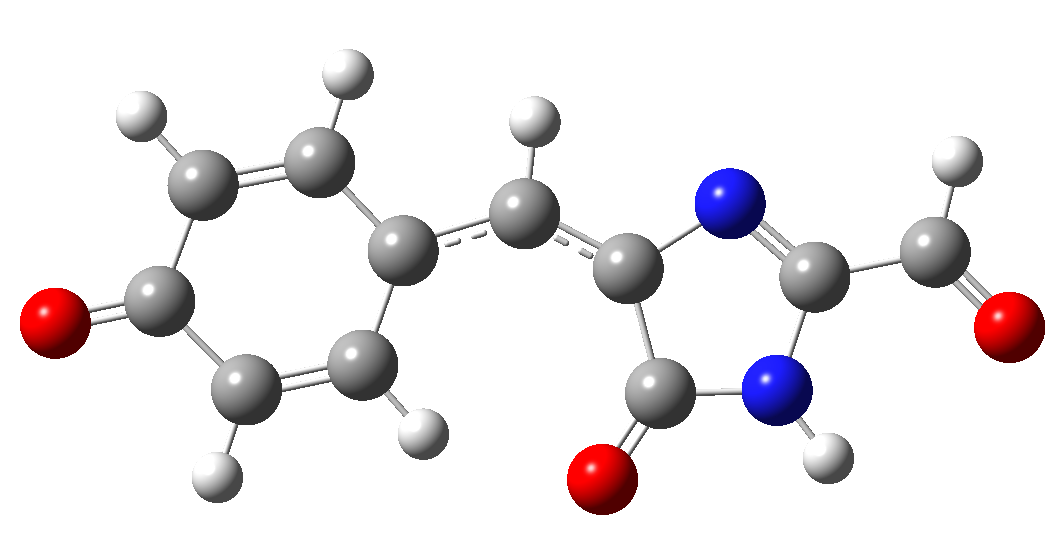


1.24

1.45

1.38

1.42

1.48

1.45

1.38

1.41

1.37

1.41

1.30

1.50

1.21

1.36

1.39

1.43

1.23

**S_1_ minimum torsion A (imidazolinone twist):**

**
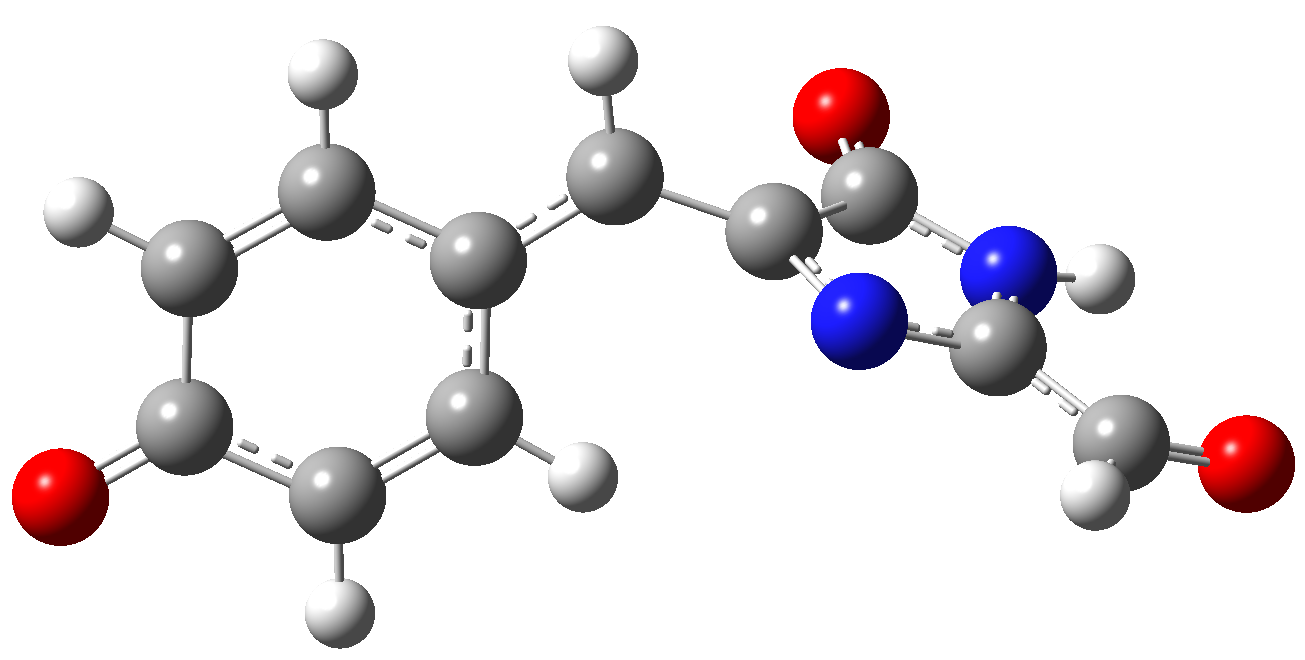
**

1.24

1.45

1.44

1.36

1.37

1.43

1.43

1.40

1.46

1.33

1.34

1.50

1.21

1.38

1.36

1.45

1.22

**S_1_ minimum torsion B (phenyl twist):**

**
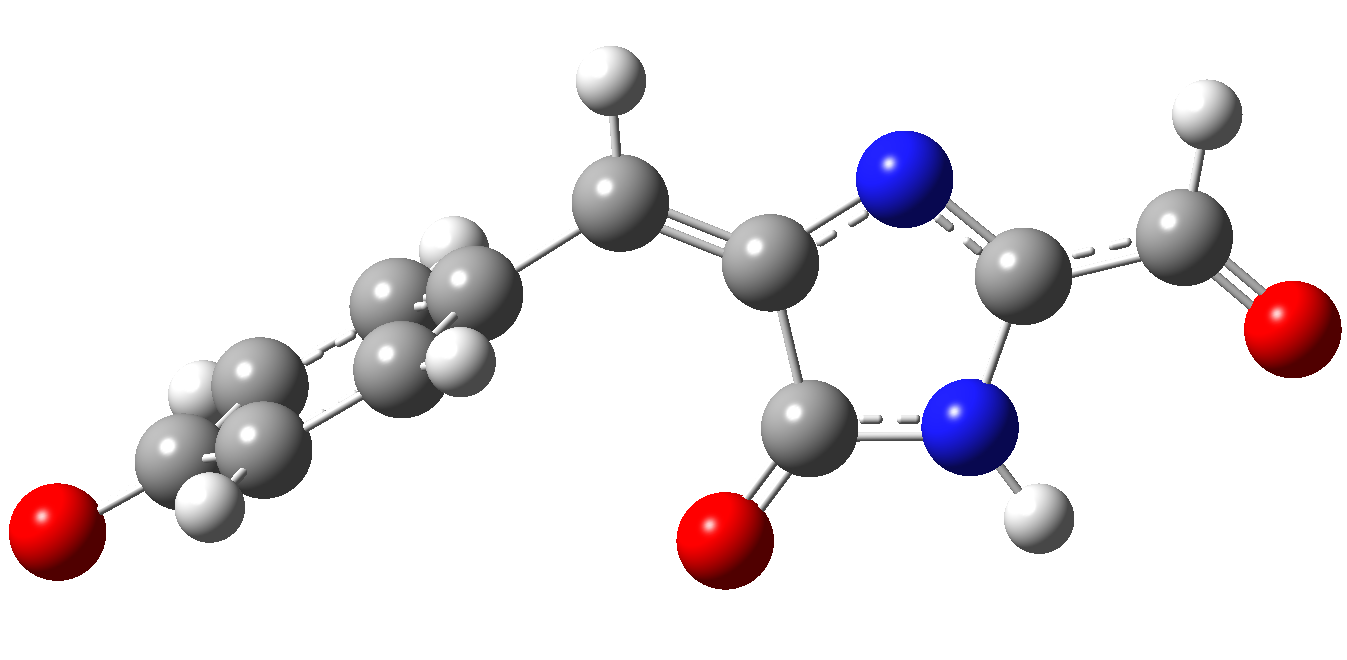
**

1.47

1.38

1.35

1.32

1.41

1.23

1.39

1.37

1.21

1.48

1.23

1.46

1.46

1.37

1.37

1.43

1.43

**S_1_/S_0_ one-bond flip MECI:**

**
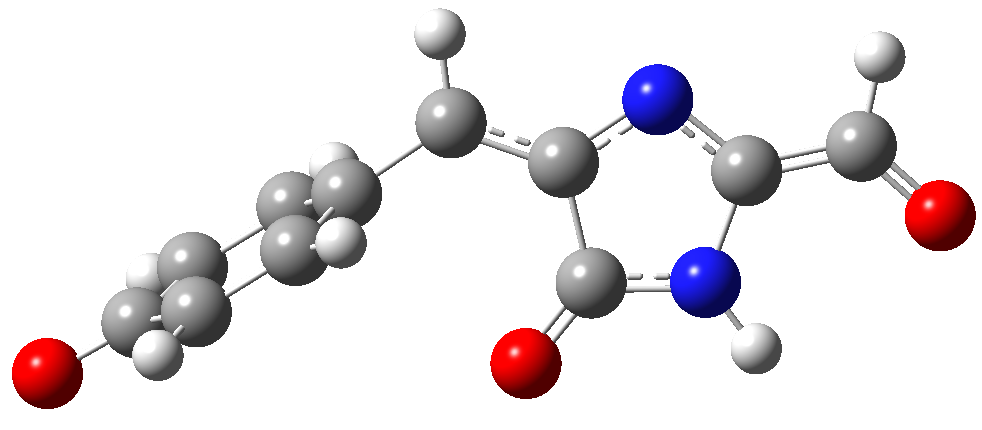
**

1.20

1.50

1.50

1.37

1.37

1.50

1.45

1.45

1.39

1.33

1.34

1.41

1.33

1.22

1.47

1.37

1.24

**Derivative coupling vector at S_1_/S_0_ MECI:**

**
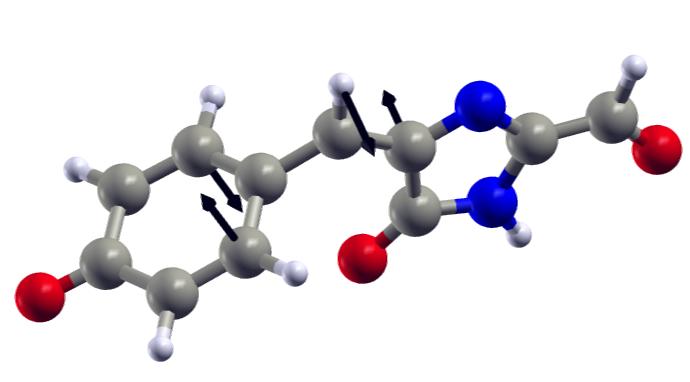
**

**Gradient difference vector at S_1_/S_0_ MECI:**

**
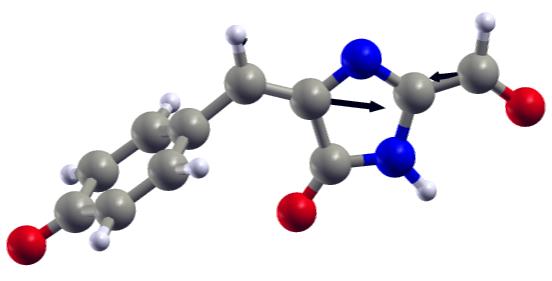
**

**S_0_ gradient at S_1_/S_0_ MECI:**

**
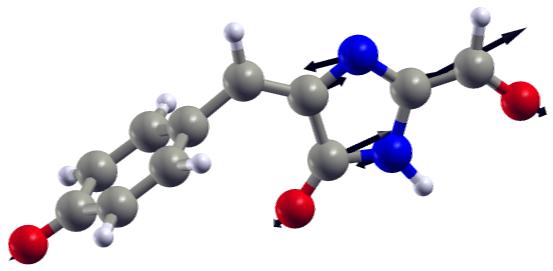
**

**S_1_ gradient at S_1_/S_0_ MECI:**

**
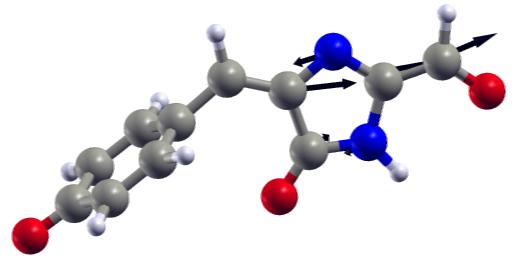
**
