# Supplementary material for: Chromophore Protonation State Controls Photoswitching of the Fluoroprotein asFP595
Source: PLoS Comput Biol. 2008 Mar 21;4(3):e1000034. doi: 10.1371/journal.pcbi.1000034 (PMC2274881; doi:10.1371/journal.pcbi.1000034)
Supplement: Figure S4 — Cis-to-trans isomerization of the neutral chromophore. (A) Chromophore (MYG) conical intersection geometry adopted during excited state QM/MM MD simulation. The CH bridge rotated upwards (i.e., towards His197). (B) Time-evolution of the ring-bridging dihedral angles A (magenta) and B (blue). (C) S0 (black) and S1 (red) potential energy traces along the QM/MM trajectory. Photon absorption brings the system into S1 (yellow area), until decay back to S0 occurs (dashed line) at the CI seam. (D,E) The hydrogen-bonding network in the chromophore cavity stayed stable during the isomerization of MYG. (8.79 MB DOC) [file pcbi.1000034.s004.doc]

**Optimized *ab initio* geometries of Z*_trans_***

**S_0_ planar minimum:**

1.21

1.47

1.34

1.46

1.37

1.43

1.37

1.47

1.35

1.46

1.46

1.21

1.38

1.36

1.33

1.21

1.43

**S_1_ planar minimum:**


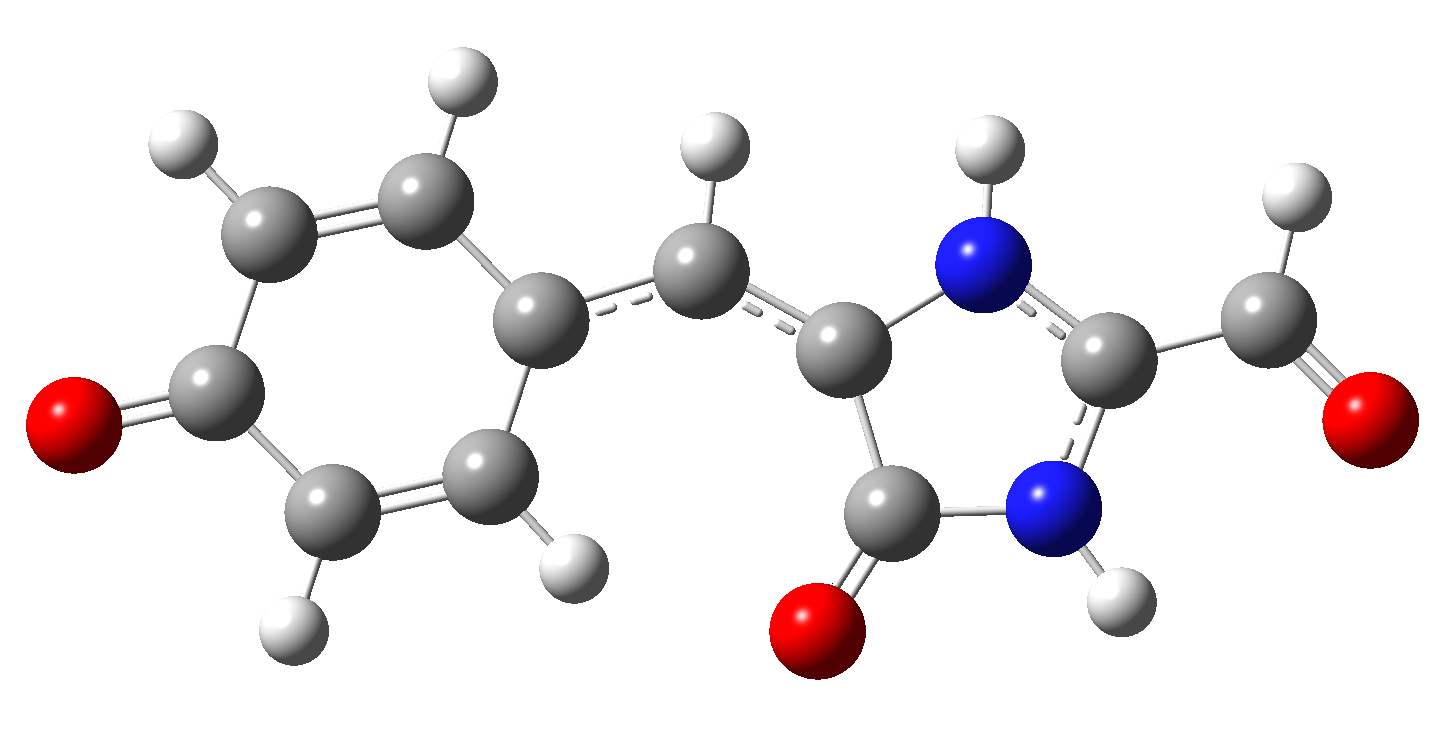


1.26

1.44

1.37

1.43

1.44

1.43

1.38

1.41

1.40

1.41

1.35

1.47

1.20

1.39

1.36

1.42

1.23

**S_1_/S_0_ hula-twist MECI:**

**
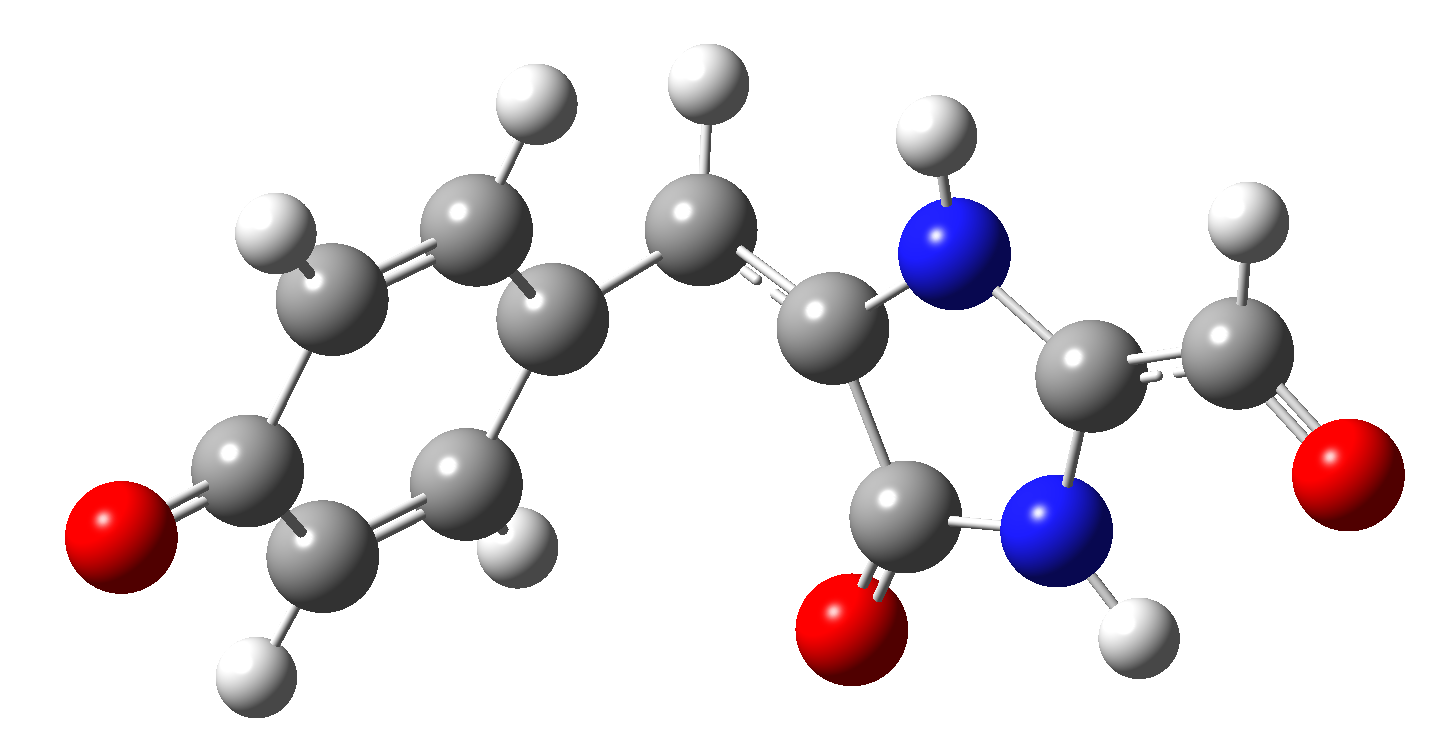
**

1.45

1.41

92º

1.22

1.47

1.47

1.35

1.35

1.45

1.45

1.40

1.39

1.51

1.38

1.38

1.40

1.23

1.20

**Derivative coupling vector at S_1_/S_0_ MECI:**

**
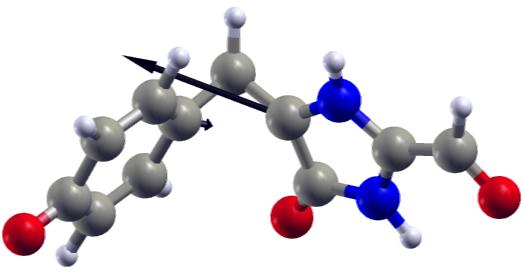
**

**Gradient difference vector at S_1_/S_0_ MECI:**

**
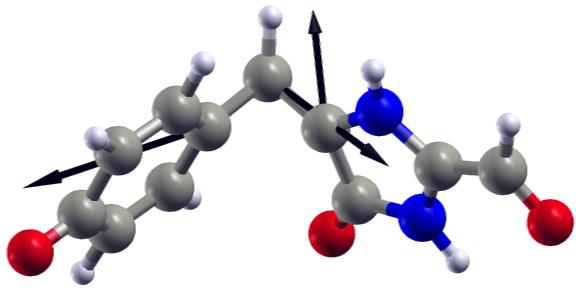
**

**S_0_ gradient at S_1_/S_0_ MECI:**

**
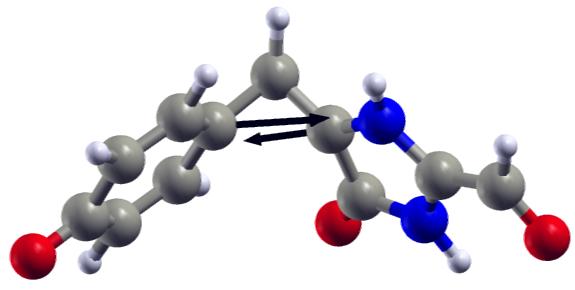
**

**S_1_ gradient at S_1_/S_0_ MECI:**

**
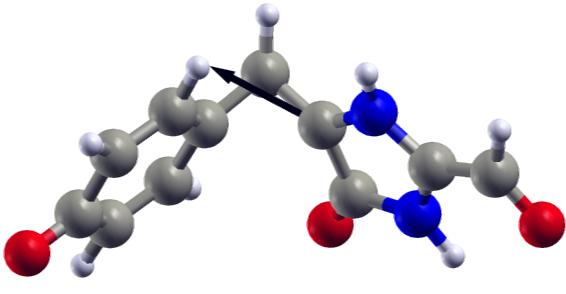
**
