# Supplementary figures and images for: Chromophore Protonation State Controls Photoswitching of the Fluoroprotein asFP595
Source: PLoS Comput Biol. 2008 Mar 21;4(3):e1000034. doi: 10.1371/journal.pcbi.1000034 (PMC2274881; doi:10.1371/journal.pcbi.1000034)

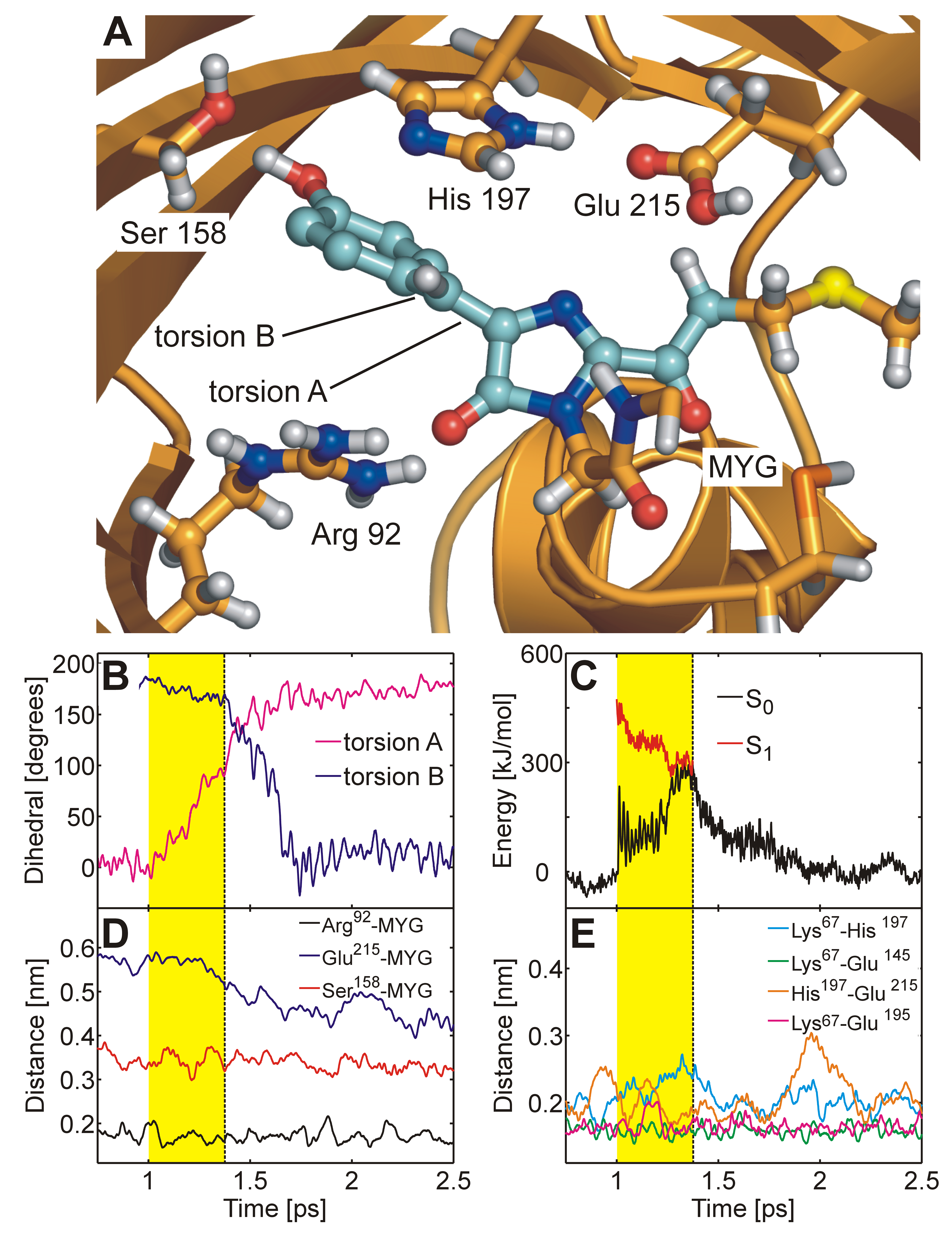

Supplement: Figure S5 — Influence of the protein environment on the deactivation of the anionic cis chromophore Acis. (A) S0 and S1 energies along a representative trajectory (run h, Table S6). The protein environment stabilized S0 and S1 (black and red lines, respectively) relative to the gas phase (dashed blue and green lines, respectively). (B) Energy difference ΔE between the protein and the gas phase for S0 (black) and for S1 (red). The dashed line indicates the decay at the CI seam. (1.30 MB TIF) [file pcbi.1000034.s005.tif]

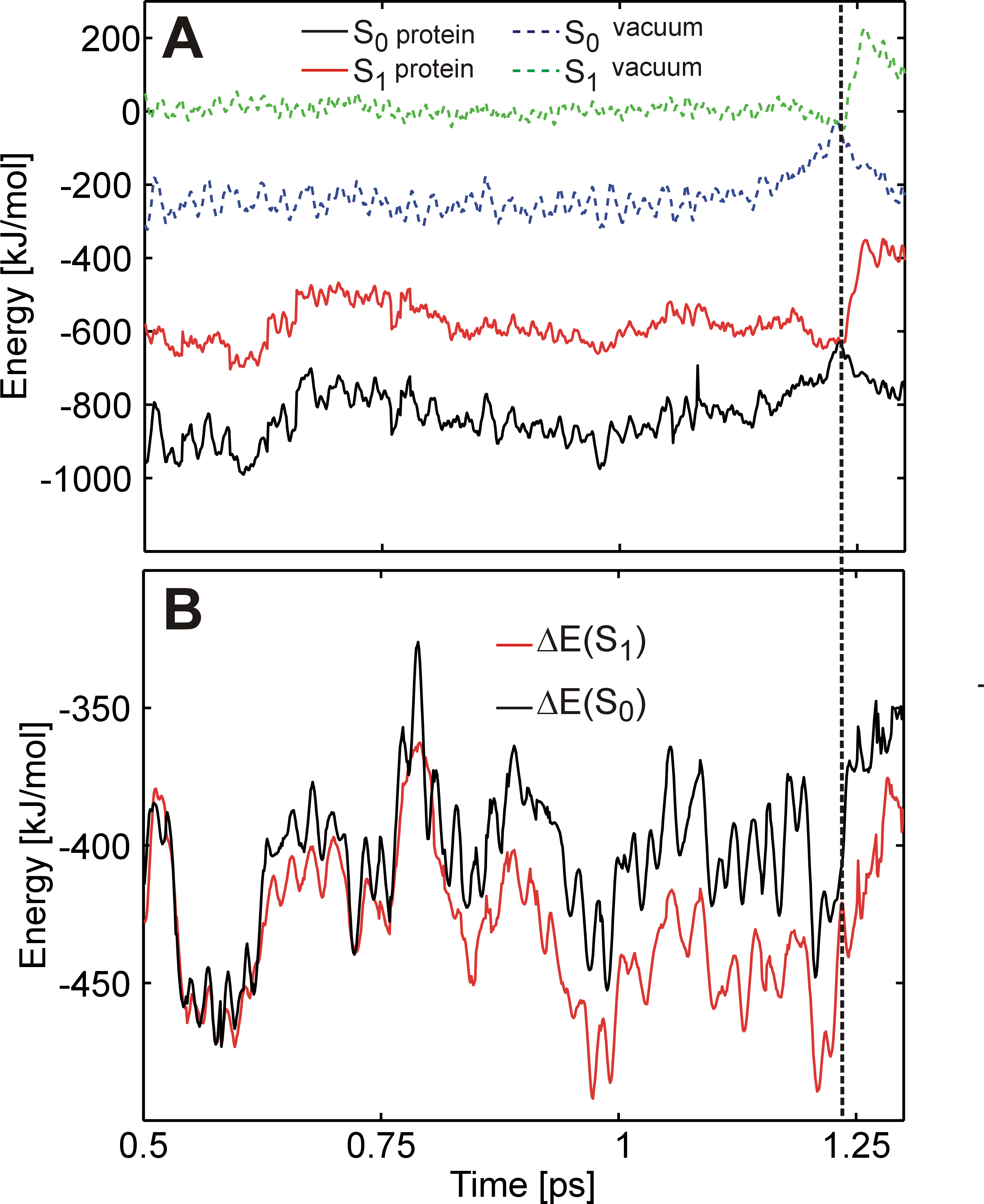

Supplement: Figure S6 — Isomerization of the cis zwitterion induced by conformational flooding. (A) Hula-twist structure adopted during isomerization trajectory (B) Ground (S0, black) and excited state (S1, red) potential energy traces along the trajectory. S0 and S1 come energetically close, but the surface crossing seam was not encountered. The surface hop was therefore imposed at the structure with the minimum energy gap (dashed black line). The time evolution of the flooding potential Vfl is shown in the inset. (C) Time evolution of the torsion angles A (magenta) and B (blue). (D) S0 and S1 energies (black and red lines, respectively) along the isomerization trajectory. The protein environment stabilizes S0 and S1 relative to the gas phase (dashed blue and green lines, respectively). The energy offset is 1.97·106 kJ/mol. (E) Energy difference ΔE between the protein and the gas phase for S0 (black) and S1 (red). S0 is stabilized slightly stronger than S1 along the whole isomerization pathway. (7.52 MB TIF) [file pcbi.1000034.s006.tif]

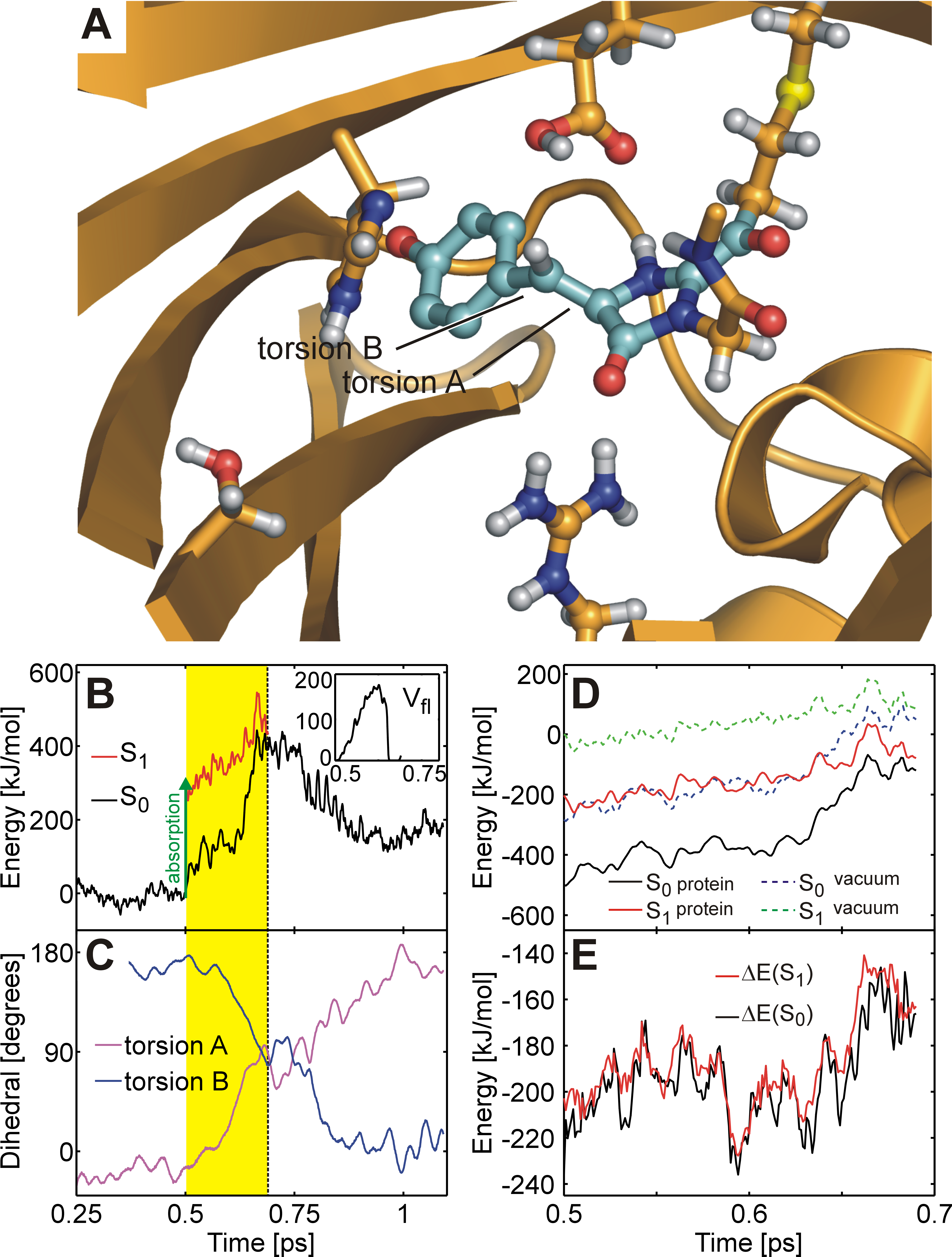

Supplement: Table S1 — RASSCF(18,7+4+5)2,2/6-31G* results on Ntrans. (0.03 MB TIF) [file pcbi.1000034.s007.tif]
