# Supplementary material for: Chromophore Protonation State Controls Photoswitching of the Fluoroprotein asFP595
Source: PLoS Comput Biol. 2008 Mar 21;4(3):e1000034. doi: 10.1371/journal.pcbi.1000034 (PMC2274881; doi:10.1371/journal.pcbi.1000034)
Supplement: Table S2 — RASSCF(18,7+4+5)2,2/6-31G* results on Atrans. (0.03 MB DOC) [file pcbi.1000034.s008.doc]

**Table S1. RASSCF(18,7+4+5)[2,2]/6-31G* results on N*_trans_*.**

| Geometry | S_0_ energy  (a.u.) | S_1_ energy  (a.u.) | S_1_ – S_0_ (kcal/mol) | ΔE(S_1_)^a^  (kcal/mol) |
| --- | --- | --- | --- | --- |
| S_0_ planar | -754.85523 | -754.67064 | 115.8 | 18.2 |
| S_1_ planar | -754.81771 | -754.69969 | 74.1 | 0.0 |
| S_1_ torsion A | -754.75642 | -754.72072 | 22.4 | -13.2 |
| S_1_ torsion B | -754.82604 | -754.68142 | 90.7 | 11.5 |
| S_1_/S_0_ MECI | -754.69460 | -754.69430 | 0.2 | 3.4 |

^a^ Relative energy to the S_1_ planar minimum energy.
