# Supplementary material for: Chromophore Protonation State Controls Photoswitching of the Fluoroprotein asFP595
Source: PLoS Comput Biol. 2008 Mar 21;4(3):e1000034. doi: 10.1371/journal.pcbi.1000034 (PMC2274881; doi:10.1371/journal.pcbi.1000034)
Supplement: Table S3 — RASSCF(18,7+4+5)2,2/6-31G* results on Ztrans. (0.03 MB DOC) [file pcbi.1000034.s009.doc]

**Table S2. RASSCF(18,7+4+5)[2,2]/6-31G* results on A*_trans_*.**

| Geometry | S_0_ energy  (a.u.) | S_1_ energy  (a.u.) | S_1_ – S_0_ (kcal/mol) | ΔE(S_1_)^a^  (kcal/mol) |
| --- | --- | --- | --- | --- |
| S_0_ planar | -754.31049 | -754.18584 | 78.2 | 7.3 |
| S_1_ planar | -754.29789 | -754.19747 | 63.0 | 0.0 |
| S_1_ torsion A | -754.25287 | -754.20354 | 31.0 | -3.8 |
| S_1_ torsion B | -754.23512 | -754.21686 | 11.5 | -12.2 |
| S_1_/S_0_ MECI^b^ | -754.20902 | -754.20900 | 0.0 | -7.2 |

^a^ Relative energy to the S_1_ planar minimum energy.

^b^ The structure is not fully optimized because of convergence difficulties. However, since the S_1_–S_0_ energy difference is zero, the reported structure is on the conical intersection hyperline near the minimum, and complete optimization can only further reduce the 5 kcal/mol energy difference to the lowest S_1_ minimum.
