# Supplementary material for: Chromophore Protonation State Controls Photoswitching of the Fluoroprotein asFP595
Source: PLoS Comput Biol. 2008 Mar 21;4(3):e1000034. doi: 10.1371/journal.pcbi.1000034 (PMC2274881; doi:10.1371/journal.pcbi.1000034)
Supplement: Table S4 — CASSCF(6,6)/3-21G results on Ztrans. (0.03 MB DOC) [file pcbi.1000034.s010.doc]

**Table S3. RASSCF(18,7+4+5)[2,2]/6-31G* results on Z*_trans_*.**

| Geometry | S_0_ energy  (a.u.) | S_1_ energy  (a.u.) | S_1_ – S_0_ (kcal/mol) | ΔE(S_1_)^a^  (kcal/mol) |
| --- | --- | --- | --- | --- |
| S_0_ planar | -754.81435 | -754.69163 | 77.0 | 11.0 |
| S_1_ planar | -754.80189 | -754.70913 | 58.2 | 0.0 |
| S_1_/S_0_ MECI | -754.68273 | -754.68271 | 0.0 | 16.6 |

^a^ Relative energy to the S_1_ planar minimum energy.
