# Supplementary material for: Chromophore Protonation State Controls Photoswitching of the Fluoroprotein asFP595
Source: PLoS Comput Biol. 2008 Mar 21;4(3):e1000034. doi: 10.1371/journal.pcbi.1000034 (PMC2274881; doi:10.1371/journal.pcbi.1000034)
Supplement: Table S5 — Cartesian coordinates of optimized structures at RASSCF(18,7+4+5)2,2/6-31G* level. (0.05 MB DOC) [file pcbi.1000034.s011.doc]

**Table S4. CASSCF(6,6)/3-21G results on Z*_trans_*.**

| Geometry | S_0_ energy  (a.u.) | S_1_ energy  (a.u.) | S_1_ – S_0_ (kcal/mol) | ΔE(S_1_)^a^  (kcal/mol) |
| --- | --- | --- | --- | --- |
| S_0_ planar | -750.479979 | -750.344619 | 84.9 | 21.1 |
| S_1_ planar | -750.463156 | -750.378208 | 53.3 | 0.0 |
| S_1_/S_0_ MECI | -750.354246 | -750.354196 | 0.0 | 15.1 |

^a^ Relative energy to the S_1_ planar minimum energy.
