# Supplementary material for: Chromophore Protonation State Controls Photoswitching of the Fluoroprotein asFP595
Source: PLoS Comput Biol. 2008 Mar 21;4(3):e1000034. doi: 10.1371/journal.pcbi.1000034 (PMC2274881; doi:10.1371/journal.pcbi.1000034)
Supplement: Table S6 — Excited state lifetimes and conformations from the MD simulations of the anionic chromophores Atrans and Acis. In runs A–J, His197 was modelled as cationic, whereas in runs K-T, His197 was modelled as neutral (singly protonated at Nδ). (0.04 MB DOC) [file pcbi.1000034.s012.doc]

**Table S5)**

**Cartesian coordinates of optimized structures at RASSCF(18,7+4+5)[2,2]/6-31G* level:**

**N*_trans_* – S_0_ minimum N*_trans_* – S_1_ planar minimum**

6 -3.840930 -1.196884 -.000584

6 -2.495272 -1.512612 .001353

6 -1.498413 -.519331 .000851

6 -1.915323 .819438 -.001281

6 -3.265422 1.140934 -.002822

6 -4.231822 .140326 -.001919

1 -4.593293 -1.963262 -.001041

1 -2.207211 -2.548940 .002563

1 -1.193344 1.608109 -.001055

1 -3.563105 2.176176 -.004065

8 -5.556794 .405237 -.004235

6 -.112230 -.983971 .001441

1 -.015388 -2.055682 .002311

6 1.093292 -.367993 .000872

6 1.516240 1.069607 .001310

7 2.261400 -1.158680 .000920

6 3.249821 -.346817 .000662

7 2.889759 .982413 .000654

1 3.513282 1.757585 .000237

8 .884302 2.088866 .002154

6 4.661591 -.770799 -.000448

8 5.561012 .029616 -.002384

1 4.828924 -1.843042 -.001543

1 -5.712274 1.339453 -.009570

6 -3.854700 -1.230326 .000080

6 -2.550853 -1.595438 .001556

6 -1.478236 -.606715 -.000914

6 -1.875941 .794800 -.005932

6 -3.197829 1.143187 -.006537

6 -4.197755 .153733 -.003460

1 -4.649690 -1.951630 .001614

1 -2.285611 -2.636632 .004316

1 -1.114904 1.549073 -.007276

1 -3.480788 2.181965 -.009065

8 -5.494819 .443268 -.003702

6 -.188342 -1.037240 .003085

1 -.027074 -2.100130 .007315

6 1.115998 -.315448 .000898

6 1.499695 1.085746 .005881

7 2.183421 -1.089825 -.002663

6 3.279578 -.288456 -.001970

7 2.875158 1.004327 .002881

1 3.480584 1.795555 .005505

8 .869542 2.133097 .009949

6 4.639005 -.734474 -.003457

8 5.586371 .024896 -.001622

1 4.771133 -1.813955 -.006554

1 -5.645141 1.380369 -.005546

**N*_trans_* – S_1_ torsion A minimum N*_trans_* – S_1_ torsion B minimum**

6 .088535 .055581 .023490

6 -.111101 .029638 1.373875

6 .999051 -.045391 2.275576

6 2.321870 -.086124 1.734187

6 2.525854 -.058554 .378433

6 1.412262 .013806 -.482211

1 -.725665 .114469 -.676519

1 -1.111844 .068902 1.775386

1 3.147356 -.127836 2.429655

1 3.523533 -.081357 -.029451

8 1.536907 .048285 -1.794174

6 .810673 -.046523 3.648114

1 -.221649 .030693 4.000492

6 1.888642 -.100096 4.630893

6 2.506554 1.043581 5.196883

7 2.329584 -1.253368 5.189996

6 3.217113 -.897308 6.077878

7 3.368321 .467082 6.117502

1 3.973479 .962776 6.726337

8 2.381344 2.233073 4.959001

6 3.945202 -1.820260 6.908658

8 4.777299 -1.494841 7.724334

1 3.685623 -2.863183 6.747843

1 2.446166 .027152 -2.081688

6 .355823 -.798721 .486764

6 .333362 -.298622 1.753900

6 1.345972 .605066 2.231896

6 2.369043 .994664 1.308477

6 2.398425 .513429 .035741

6 1.398121 -.406542 -.384939

1 -.390420 -1.494573 .146471

1 -.434844 -.588588 2.443375

1 3.127980 1.667271 1.657397

1 3.165095 .782409 -.665372

8 1.493757 -.854854 -1.611434

6 1.326473 1.103965 3.615198

1 .905333 2.075891 3.798685

6 1.891826 .338550 4.627871

6 2.475441 -.983496 4.374661

7 1.993171 .642179 5.940789

6 2.587487 -.384312 6.520112

7 2.889230 -1.382196 5.605866

1 3.341846 -2.240345 5.822049

8 2.568553 -1.591983 3.317053

6 2.902438 -.499756 7.913320

8 3.451818 -1.472932 8.397840

1 2.609594 .350185 8.525186

1 .817102 -1.487010 -1.828011

**N*_trans_* – S_1_/S_0_ MECI A*_trans_* – S_0_ minimum**

6 -3.842597 -.826396 .389946

6 -2.625074 -1.373284 .762975

6 -1.419496 -.847414 .251087

6 -1.496055 .261479 -.652562

6 -2.709327 .803308 -1.026546

6 -3.889184 .257423 -.506557

1 -4.770862 -1.221285 .773118

1 -2.607231 -2.214049 1.444919

1 -.570745 .671113 -1.044030

1 -2.745150 1.636392 -1.716579

8 -5.112704 .716670 -.839794

6 -.123459 -1.429379 .463929

1 .000122 -2.443433 .863675

6 1.105229 -.678853 .114169

6 1.432378 -.103335 1.284382

7 2.051019 -.540243 -.824893

6 2.931838 .233153 -.240440

7 2.595448 .559127 1.052929

1 3.174609 1.085039 1.659578

8 .658865 -.313943 2.264380

6 4.122982 .732300 -.867624

8 4.890242 1.469543 -.302639

1 4.275072 .399043 -1.884549

1 -5.067119 1.456768 -1.434497

6 -3.826167 -1.257263 .000264

6 -2.506729 -1.548590 .002578

6 -1.470934 -.535981 .002264

6 -1.933106 .837103 -.000073

6 -3.250518 1.148306 -.002611

6 -4.299017 .128089 -.002996

1 -4.575633 -2.029662 .000414

1 -2.196916 -2.581398 .004699

1 -1.196338 1.615890 .000426

1 -3.578031 2.173512 -.004491

8 -5.489658 .408592 -.006063

6 -.155164 -.953091 .002767

1 -.029718 -2.024507 .003451

6 1.105782 -.315306 .002006

6 1.508833 1.082294 .003047

7 2.231265 -1.112462 .000079

6 3.262870 -.324342 -.000310

7 2.888602 .999947 .001611

1 3.498363 1.783400 .001610

8 .896642 2.136844 .005216

6 4.629773 -.782944 -.003527

8 5.600487 -.054635 -.004298

1 4.737685 -1.866942 -.005186

**A*_trans_* – S_1_ planar minimum A*_trans_* – S_1_ torsion A minimum**

6 -3.869181 -1.210274 -.010890

6 -2.527888 -1.517125 -.008553

6 -1.518734 -.524698 .003811

6 -1.944205 .824110 .014613

6 -3.281272 1.155820 .012009

6 -4.328224 .159896 -.000495

1 -4.615105 -1.985503 -.019764

1 -2.232231 -2.553466 -.015845

1 -1.210562 1.603110 .023988

1 -3.585820 2.187667 .020047

8 -5.533408 .456058 -.001932

6 -.119446 -1.003775 .008349

1 -.037289 -2.077077 .020322

6 1.107796 -.405686 .001173

6 1.533629 1.034679 -.014909

7 2.270612 -1.202392 .009643

6 3.285371 -.382874 .001372

7 2.891352 .950893 -.013688

1 3.516871 1.722552 -.022083

8 .893921 2.058527 -.026138

6 4.679311 -.717280 .005871

8 5.572239 .134583 -.002901

1 4.904636 -1.780892 .016877

6 .104740 .110103 -.006600

6 -.070191 .109311 1.345193

6 1.009292 -.016961 2.279391

6 2.300262 -.145788 1.679481

6 2.492180 -.145287 .325434

6 1.409601 -.016165 -.621056

1 -.736261 .205544 -.668344

1 -1.065408 .206163 1.741532

1 3.156796 -.262189 2.311359

1 3.478989 -.250332 -.084892

8 1.577319 -.017226 -1.846140

6 .766323 -.018956 3.654685

1 -.245112 .066118 3.986217

6 1.788303 -.091669 4.687828

6 2.417566 1.084512 5.382262

7 2.319740 -1.213014 5.178583

6 3.232970 -.864758 6.095328

7 3.313102 .487432 6.248566

1 3.937376 .971913 6.855666

8 2.224079 2.270266 5.277108

6 4.046904 -1.810563 6.828290

8 4.863663 -1.464255 7.659003

1 3.880620 -2.859243 6.586638

**A*_trans_* – S_1_ torsion B minimum A*_trans_* – S_1_/S_0_ MECI**

6 .275917 -.708042 .331388

6 .312424 -.342610 1.647759

6 1.373968 .450662 2.180049

6 2.415367 .850412 1.288288

6 2.406420 .501324 -.033011

6 1.323867 -.297134 -.595037

1 -.526087 -1.314338 -.064072

1 -.465786 -.657978 2.325601

1 3.226434 1.434598 1.695035

1 3.201104 .800071 -.701167

8 1.292113 -.600767 -1.788449

6 1.372829 .878572 3.589796

1 .892281 1.800722 3.837744

6 1.950279 .180469 4.631859

6 2.659031 -1.117576 4.507165

7 1.955861 .571998 5.927160

6 2.598357 -.349992 6.621093

7 3.034749 -1.381593 5.793832

1 3.535212 -2.178044 6.105575

8 2.873383 -1.809619 3.535690

6 2.834216 -.333839 8.009911

8 3.437368 -1.202318 8.633916

1 2.436150 .539333 8.517348

**Z*_trans_* – S_0_ minimum Z*_trans_* – S_1_ planar minimum**

6 -3.850368 -1.258382 .000833

6 -2.544340 -1.573814 -.000177

6 -1.491035 -.560166 -.001694

6 -1.923687 .833676 -.002274

6 -3.229604 1.161160 -.001191

6 -4.292526 .145246 .002673

1 -4.616233 -2.011901 .001585

1 -2.246538 -2.608185 .000308

1 -1.176667 1.600357 -.002883

1 -3.545876 2.188010 -.001572

8 -5.469228 .442890 .004256

6 -.194883 -.999397 -.001961

1 -.082625 -2.072602 -.000708

6 1.069922 -.326509 -.002146

6 1.505752 1.068273 -.001430

7 2.218535 -1.064034 -.001342

6 3.320139 -.311246 .000557

1 2.234666 -2.060555 -.001170

7 2.885606 .973906 .001125

1 3.489423 1.766721 .003359

8 .893941 2.107862 .000368

6 4.683600 -.756776 .001372

8 5.614078 .016065 .002179

1 4.837252 -1.834818 -.000068

8 1.583704 .288802 .000000

8 2.659205 -4.733065 .000000

8 -1.281520 5.080166 .000000

6 1.465172 -4.391960 .000000

6 1.002404 -3.104754 .000000

6 -.217896 -1.346833 .000000

6 1.173515 -.861445 .000000

6 -1.313853 -.485028 .000000

6 -1.242427 .992776 .000000

6 -1.218836 1.735438 1.249841

6 -1.218836 3.100433 1.280200

6 -1.239486 3.876709 .000000

6 -1.218836 3.100433 -1.280200

6 -1.218836 1.735438 -1.249841

7 -.264571 -2.673645 .000000

7 1.876081 -1.995035 .000000

1 .679709 -5.135353 .000000

1 2.861503 -2.079130 .000000

1 -2.279675 -.934108 .000000

1 -1.183246 1.152069 2.163453

1 -1.189622 3.665378 2.206005

1 -1.189622 3.665378 -2.206005

1 -1.183246 1.152069 -2.163453

6 -3.884228 -1.219891 .000670

6 -2.554183 -1.542053 .003246

6 -1.538176 -.541681 .002158

6 -1.936485 .812430 -.001321

6 -3.277904 1.153529 -.004267

6 -4.301378 .155533 -.003638

1 -4.645608 -1.977503 .001165

1 -2.264425 -2.578541 .005853

1 -1.196641 1.585236 -.002216

1 -3.583432 2.183164 -.007497

8 -5.517647 .467460 -.007086

6 -.171749 -1.005036 .003604

1 -.082719 -2.078688 .003483

6 1.068381 -.361382 .003808

6 1.521227 1.033782 .007208

7 2.249635 -1.129961 .001781

6 3.347220 -.336237 .000647

1 2.281113 -2.122672 -.003288

7 2.908340 .952283 .004540

1 3.516595 1.740479 .004238

8 .909794 2.065508 .009863

6 4.710201 -.721980 -.004734

8 5.604689 .118148 -.008571

1 4.926683 -1.786147 -.009645

**Z*_trans_* – S_1_/S_0_ MECI**

6 -1.263083 2.351693 -.102303

6 -1.201508 1.056885 .275201

6 -.366231 .112475 -.433499

6 .364802 .590870 -1.592513

6 .306018 1.877572 -1.990651

6 -.495043 2.860924 -1.247723

1 -1.897163 3.053336 .407161

1 -1.783043 .700996 1.102903

1 .959675 -.115983 -2.149102

1 .846921 2.230494 -2.848722

8 -.524083 4.031283 -1.573065

6 -.301341 -1.305993 -.117018

1 -.200946 -2.027344 -.903359

6 .758632 -1.116967 .788840

6 .696759 -.691542 2.238304

7 2.067311 -1.584754 .610456

6 2.812902 -1.477886 1.781882

1 2.447697 -1.849646 -.261738

7 1.962220 -.934696 2.719394

1 2.227476 -.802210 3.670085

8 -.230988 -.275452 2.870105

6 4.151050 -1.836689 2.012121

8 4.685092 -1.671763 3.105361

1 4.693142 -2.268827 1.176713
