# Supplementary material for: Chromophore Protonation State Controls Photoswitching of the Fluoroprotein asFP595
Source: PLoS Comput Biol. 2008 Mar 21;4(3):e1000034. doi: 10.1371/journal.pcbi.1000034 (PMC2274881; doi:10.1371/journal.pcbi.1000034)
Supplement: Text S1 — Ab initio calculations in the gas phase. (0.03 MB DOC) [file pcbi.1000034.s013.doc]

**Table S6) Ultra-fast radiationless deactivation of the anionic chromophores**

| Run | τ(S_1_) [ps] | initial conformation | final conformation |
| --- | --- | --- | --- |
| A | 0.363 | trans | trans |
| B | 0.483 | trans | trans |
| C | 1.190 | trans | trans |
| D | 0.461 | trans | trans |
| E | 0.508 | trans | trans |
| F | 3.141 | cis | cis |
| G | 1.997 | cis | cis |
| H | 0.731 | cis | cis |
| I | 2.811 | cis | cis |
| J | 3.451 | cis | cis |
| K | 0.581 | trans | trans |
| L | 0.565 | trans | trans |
| M | 0.493 | trans | trans |
| N | 0.837 | trans | trans |
| O | 0.300 | trans | trans |
| P | 3.743 | cis | cis |
| Q | 4.900 | cis | cis |
| R | 1.176 | cis | cis |
| S | 2.965 | cis | cis |
| T | 1.750 | cis | cis |

**Table S6.** Excited state lifetimes and conformations from the MD simulations of the anionic chromophores *A_trans_* and *A_cis_*. In runs A-J, His197 was modelled as cationic, whereas in runs K-T, His197 was modelled as neutral (singly protonated at N_δ_).
