# Supplementary material for: Chromophore Protonation State Controls Photoswitching of the Fluoroprotein asFP595
Source: PLoS Comput Biol. 2008 Mar 21;4(3):e1000034. doi: 10.1371/journal.pcbi.1000034 (PMC2274881; doi:10.1371/journal.pcbi.1000034)
Supplement: Text S2 — Influence of the π-stacked Histidine 197. (0.02 MB DOC) [file pcbi.1000034.s014.doc]

# Text S1) Ab initio calculations in the gas phase

# Ab initio calculations of the trans chromophore of asFP595 were performed in the gas phase using the restricted active space self-consistent field (RASSCF) method.**[[1]](#footnote-2)**,**[[2]](#footnote-3)** The *π* system of the chromophore of asFP595 is made up of 18 electrons and 16 orbitals. Performing a full complete active space self-consistent field (CASSCF) calculation in such a large active space would require considerable computational effort due to the very large number of configurations. To make the computations feasible, we have used the RASSCF approach in which the excitations in the wave function are restricted. This reduction is done by subdividing the active space into three categories: a set of orbitals with a limited number of vacancies (called the RAS1 space), a fully active orbital set (RAS2), and a set of orbitals with a limited number of electrons (RAS3). We have restricted the excitations from the RAS1 space to singles and doubles only, and allowed only two electrons at most in RAS3. The choice of the molecular orbitals making up the different restricted active spaces is critical. The idea is to include the most important orbitals for the electron correlation in the RAS2 space, where no restriction in the excitations is imposed. It has already been shown in a previous study that this method can be used to reproduce CASSCF results with a good accuracy, provided a good RAS2 partition is chosen.**[[3]](#footnote-4)**

# The abbreviation (*n*,*m*) is used in this article to define the number of active electrons *n* and active orbitals *m*. The notation (*n*,*m*^I^+*m*^II^+*m*^III^)[*h*,*e*] is used for specifying the type of RASSCF, with *m*^I^, *m*^II^, and *m*^III^ defining the number of active orbitals in RAS1, RAS2, and RAS3, respectively. The values of *h* and *e* specify the number of holes in RAS1 and electrons in RAS3, respectively, thus defining the restriction of the excitations in these subspaces.

# For the asFP595 chromophore, we found that the most cost effective (i.e., best ratio accuracy/cost) level of calculation is RASSCF(18,7+4+5)[2,2]/6-31G*, including all the 18 *π* electrons and the 16 *π* orbitals in the active space, and using 4 orbitals (HOMO-1, HOMO, LUMO, LUMO+1) in the RAS2 space. The energies and structures of all the critical points found at this level of calculations are displayed in the following tables and figures for each of the three protonation state of the chromophore (neutral “N”, anionic “A”, and zwitterionic “Z”). An attempt to enlarge the RAS2 space to 6 orbitals did not change the results sensibly, while increasing substantially the computational effort.

# The results for the neutral chromophore of asFP595, N*_trans_*, are shown in Table S1 and in the figures following Table S1. The most important result is that the lowest excited state minimum found is a minimum with a torsion of the imidazolinone ring (S_1_ minimum torsion A), thus this minimum is twisted along the *trans*-to-*cis* isomerization coordinate. In the vicinity of this minimum lies a minimum energy conical intersection (S_1_/S_0_ MECI). The two degeneracy-lifting coordinates (derivative coupling and gradient difference vectors) do not involve torsion A, which implies that a crossing “seam” or degeneracy hyperline exists along the torsion A coordinate. The low energy of the MECI and the extended nature of the associated crossing seam indicate that the seam can easily be accessed for a wide range of imidazolinone twist angles. Indeed, in our excited state MD simulations we observed decay at this conical intersection back to the ground state, where the system either completed torsion A rotation to form the *cis* isomer N*_cis_*, or returned back to the initial *trans* isomer N*_trans_*, depending on which part of the extended seam was accessed.

# The results for the anionic chromophore of asFP595, A*_trans_*, are shown in Table S2 and in the figures following Table S2. Unlike the N*_trans_* global S_1_ minimum, the A*_trans_* lowest energy S_1_ minimum has a torsion of the phenyl ring (S_1_ minimum torsion B), explaining why this reaction path was favored over the *trans*-to-*cis* isomerization (torsion A) coordinate in our MD simulations. Moreover, a nearby conical intersection (S_1_/S_0_ MECI) lies just 5 kcal/mol above this minimum. In this MECI structure, the phenoxy group is orthogonal to the rest of the chromophore. The derivative coupling vector involves rotation around torsion B, indicating that twisting of the phenyl ring is needed to reach the MECI. However, the amplitude of this vector is small, thus the two electronic states may remain close in energy along torsion B, allowing the system to decay at various phenyl twist angles. Because of the low-lying energy of the MECI and the extended nature of the seam due to the small nonadiabatic coupling, the system ~~to~~ decayed at this conical intersection back to the ground state in our MD simulations. After the decay, the initial *trans* isomer was reformed in all cases.

# The results for the zwitterionic chromophore of asFP595, Z*_trans_*, are displayed in Table S3 and in the figures following Table S3. In contrast with N*_trans_* and A*_trans_*, no twisted minimum was found along torsion A or B. Only a planar S_1_ minimum could be located. The MECI does not involve a single bond rotation like in the previous cases, but rather it requires a simultaneous rotation around both torsion angles A and B, called “hula-twist” motion. The fact that there are no S_1_ minima in the region of the MECI explains why the zwitterionic chromophores were not driven towards that region of the potential energy surface during our MD simulations. Moreover, in this protonation state, the MECI lies at higher energy than the Franck-Condon energy. Thus, the “hula-twist” reaction path is not favorable energetically. We therefore expect the system to oscillate for a long time in the S_1_ planar minimum, from which the system can decay back to the ground state through fluorescence.

# In conclusion, the gas phase calculations suggest that the neutral protonation species is a photoisomerization state, the anionic protonation species is a photostabilization state, and the zwitterionic protonation species is a fluorescent state. These results were confirmed by our QM/MM excited state molecular dynamics simulations, in which the CASSCF(6,6)/3-21G level used for the chromophoric moiety was calibrated against the RASSCF calculations (e.g., compare Tables S3 and S4).

1. Olsen, J.; Roos, B. O.; Jørgensen, P.; Jensen, H. J. Aa. *J. Chem. Phys.* 89, **1988**, 2185–2192. [↑](#footnote-ref-2)
2. Malmqvist, P.-Å.; Rendell, A.; Roos, B. O. *J. Phys. Chem.* 94, **1990**, 5477–5482. [↑](#footnote-ref-3)
3. Boggio-Pasqua, M.; Robb, M. A.; Bearpark, M. J. *J. Phys. Chem. A* 109, **2005**, 8849–8856. [↑](#footnote-ref-4)
