# Supplementary material for: Chromophore Protonation State Controls Photoswitching of the Fluoroprotein asFP595
Source: PLoS Comput Biol. 2008 Mar 21;4(3):e1000034. doi: 10.1371/journal.pcbi.1000034 (PMC2274881; doi:10.1371/journal.pcbi.1000034)
Supplement: Text S3 — Conformational flooding simulations of the zwitterionic chromophores. (0.02 MB DOC) [file pcbi.1000034.s015.doc]

**Text S2) Influence of the π-stacked Histidine 197**

Due to its close proximity to the chromophore and its co-planar orientation, we expected the charge state of His197 to influence the excited state properties of the asFP595 chromophore. Recent Poisson-Boltzmann electrostatics calculations have shown that slight structural fluctuations in the local environment change the preferred protonation of the His197 imidazole ring between cationic and neutral and that both protonation states are populated at room temperature (Schäfer, L.V., et al. *Angew. Chem. Int. Ed.* 46, **2007**, 530–536).

In all our simulations of the anionic and zwitterionic chromophores, we therefore considered both states of the His197, i.e., we ran the same number of trajectories with a cationic and with a neutral imidazole ring (see Table S5). In all cases, the His197 protonation state did not influence the decay mechanism. Furthermore, we found that the S_1_ lifetimes were hardly affected. For *A_trans_*, decay times of τ = 0.46 ps (σ_+_ = 0.28 ps, σ- = 0.17 ps) and τ = 0.45 ps (σ_+_ = 0.27 ps, σ- = 0.17 ps) were obtained from the simulations with a cationic and a neutral His197, respectively. For *A_cis_*, the respective decay times were τ = 1.60 ps (σ_+_ = 0.96 ps, σ- = 0.60 ps) and τ = 2.02 ps (σ_+_ = 1.21 ps, σ- = 0.76 ps). Closer analysis of the hydrogen bonds in the chromophore cavity revealed the reason for the similar behavior. The protonated N_ε_ of the cationic His197 imidazole ring formed a hydrogen bond to the anionic Glu145 side chain, which reduced the positive charge density due to the cationic His197 above the phenoxy-ring of MYG. In case of the neutral His197, a hydrogen bond between the deprotonated N_ε_ of His197 and the cationic ammonium group of Lys67 was established, leading to the location of the positive Lys67 charge above the chromophore. Thus, in both cases, the electrostatic environment of the chromophore is similar, explaining the similar excited state lifetimes.
