# Supplementary material for: Chromophore Protonation State Controls Photoswitching of the Fluoroprotein asFP595
Source: PLoS Comput Biol. 2008 Mar 21;4(3):e1000034. doi: 10.1371/journal.pcbi.1000034 (PMC2274881; doi:10.1371/journal.pcbi.1000034)
Supplement: Text S4 — Diabatic Surface Hopping: Theory and Implementation. (0.06 MB DOC) [file pcbi.1000034.s016.doc]

**Text S3) Conformational flooding simulations of the zwitterionic chromophores**

Figure S6 shows the results obtained from conformational flooding simulations of *Z_cis_*. Similar results were obtained for *Z_trans_* (data not shown). Starting in the planar S_1_ cis minimum, the flooding potential V_fl_ induced an isomerization in the S_1_ state to the trans conformation within less than 0.5 ps. During the isomerization, the S_1_/S_0_ energy gap decreased significantly (Figure S6, b), but no spontaneous surface crossing back to S_0_ was observed. Figure S6, a) shows the structure along an isomerization trajectory at which the S_1_/S_0_ energy gap was minimal. The chromophore adopted a hula-twist conformation with the ring-bridging CH group pointing upwards (towards His197). In the hula-twist mechanism, both ring-bridging torsion angles rotated simultaneously, as shown in Figure S6, c. After excitation to S_1_, the flooding potential V_fl_ (inset of b) induced the crossing of the S_1_ barrier that separates the planar minimum from the hula-twisted conformation. Upon the barrier crossing, the chromophore escaped the influence of the flooding potential and V_fl_ dropped to (almost) zero. We imposed a surface hop at the structure with the minimal S_1_/S_0_ energy gap and switched off the flooding potential to allow an unperturbed relaxation on the S_0_ surface.

As an additional test for our assignment of the zwitterion to be the fluorescent species, we have carried out three excited state QM/MM simulations in which we applied a constant flooding potential (1 ps, V_fl_ = 100 kJ/mol). From these simulations, we obtained an expected acceleration according to α = exp[ΔF_0_/k_B_T] with ΔF_0_ = - k_B_T ln <exp[-V_fl_/k_B_T]>, where the angle brackets denote an appropriate time-average (data not shown). The acceleration thus obtained is a lower bound for the estimated decay time, in particular for the case at hand, where we must assume that the CI is accessed in a two-step process involving (i) chromophore isomerization and (ii) skeletal deformation. From the additional constant flooding simulations, we estimate a lower bound for the decay time of about 1 ns, which is in qualitative agreement with the measured fluorescence lifetime of 2.2 ns.
