# Supplementary material for: Chromophore Protonation State Controls Photoswitching of the Fluoroprotein asFP595
Source: PLoS Comput Biol. 2008 Mar 21;4(3):e1000034. doi: 10.1371/journal.pcbi.1000034 (PMC2274881; doi:10.1371/journal.pcbi.1000034)
Supplement: Video S1 — cis-to-trans isomerization of neutral chromophore. (24.36 MB DOC) [file pcbi.1000034.s017.doc]

**Text S4) Diabatic Surface Hopping: Theory and Implementation**

Following the Landau-Zener model (see M. Desouter-Lecomte, J.C. Lorquet, *J. Chem. Phys.* 71, 4391, 1979), in one dimension, the probability of decay can be written as:

,

where

,

with

,

and *Q*, the nuclear reaction coordinate. Thus:

.

In a classical trajectory one must decide when to switch from state 2 to state 1, using the gradients of state 2. If the vibrational motion of the nuclei is treated quantum mechanically, then in regions of strong coupling (large ), part of the population will transfer to the other state and part will remain on the old state. For classical trajectories this implies spawning another trajectory. When single trajectories are computed using an ab initio wavefunction to compute forces, we need a procedure for making a binary decision (hop or no hop).

To make this decision in a classical simulation, one has to monitor . For a two-state problem and a small time step Δt to integrate the classical equations of motion for the nuclei, we have:

Using

$\langle\Psi_{1}|\Psi_{2}\left(t_{1}\right)\rangle=-\theta\left(t\right)$,

we compute $\langle\Psi_{1}|\Psi_{2}\left(t_{1}\right)\rangle$ as a numerical approximation for to decide on hopping.

In addition, we monitor the energy gap $\Delta E$ between the states, which can be combined with $\langle\Psi_{1}|\Psi_{2}\left(t_{1}\right)\rangle$ to compute the probability. Thus, we could spawn two trajectories with different probabilities. However, this is not feasible in practice due to the enormous computational costs associated with spawning multiple CASSCF QM/MM trajectories. Instead, we restrict hops to the situations where the probability approaches unity. By choosing this limiting case, we will underestimate the population transfer and we only hop at the conical intersection seam. Because of the extended nature of the intersection seam, as discussed above, our trajectories do encounter such regions of high transfer probability. Our approach can yield mechanistic insights and, in the limit of a sufficiently high number of trajectories, can be used to estimate lifetimes, in particular in the case of fast processes, which are dominated by events with a very large decay probability.

We use the symbol to refer to the CASSCF configuration interaction eigenvector for state K at MD step i (K = 1 for the ground state and K = 2 for the excited state). Initially, is used to compute the gradient. The inner product represents $\langle\Psi_{1}|\Psi_{2}\left(t_{1}\right)\rangle$ and is used to monitor whether a hop has occurred. If this inner product is sufficiently small and the other inner product approaches one, then the trajectory has passed a point of an avoided or a real crossing. Provided the energy difference is less than the threshold (0.05 a.u.), we assume that the step (i-1 → i) has passed a point of real crossing, and select the eigenvector rather than to compute the gradient at step i. Energy and momentum are obviously conserved, as the trajectory never leaves the diabatic surface.
